# Angiopoietin 1 and integrin beta 1b are vital for zebrafish brain development

**DOI:** 10.3389/fncel.2023.1289794

**Published:** 2024-01-03

**Authors:** Yu-Chia Chen, Tomás A. Martins, Valentina Marchica, Pertti Panula

**Affiliations:** ^1^Department of Anatomy, University of Helsinki, Helsinki, Finland; ^2^Zebrafish Unit, Helsinki Institute of Life Science (HiLIFE), Helsinki, Finland

**Keywords:** neurovascular, neurogenesis, GABA, dopamine, histamine, hypothalamus

## Abstract

**Introduction:**

Angiopoietin 1 (angpt1) is essential for angiogenesis. However, its role in neurogenesis is largely undiscovered. This study aimed to identify the role of angpt1 in brain development, the mode of action of angpt1, and its prime targets in the zebrafish brain.

**Methods:**

We investigated the effects of embryonic brain angiogenesis and neural development using qPCR, *in situ* hybridization, microangiography, retrograde labeling, and immunostaining in the *angpt1*^*sa*14264^, *itgb1b*^*mi*371^, *tek*^*hu*1667^ mutant fish and transgenic overexpression of *angpt1* in the zebrafish larval brains.

**Results:**

We showed the co-localization of angpt1 with *notch*, *delta*, and *nestin* in the proliferation zone in the larval brain. Additionally, lack of *angpt1* was associated with downregulation of *TEK tyrosine kinase, endothelial* (*tek*), and several neurogenic factors despite upregulation of *integrin beta 1b* (*itgb1b*), *angpt2a*, *vascular endothelial growth factor aa* (*vegfaa*), and glial markers. We further demonstrated that the targeted *angpt1*^*sa*14264^ and *itgb1b*^*mi*371^ mutant fish showed severely irregular cerebrovascular development, aberrant hindbrain patterning, expansion of the radial glial progenitors, downregulation of cell proliferation, deficiencies of dopaminergic, histaminergic, and GABAergic populations in the caudal hypothalamus. In contrast to *angpt1*^*sa*14264^ and *itgb1b*^*mi*371^ mutants, the *tek*^*hu*1667^ mutant fish regularly grew with no apparent phenotypes. Notably, the neural-specific *angpt1* overexpression driven by the *elavl3 (HuC)* promoter significantly increased cell proliferation and neuronal progenitor cells but decreased GABAergic neurons, and this neurogenic activity was independent of its typical receptor *tek*.

**Discussion:**

Our results prove that *angpt1* and *itgb1b*, besides regulating vascular development, act as a neurogenic factor via notch and wnt signaling pathways in the neural proliferation zone in the developing brain, indicating a novel role of dual regulation of *angpt1* in embryonic neurogenesis that supports the concept of angiopoietin-based therapeutics in neurological disorders.

## 1 Introduction

A functional neural orchestra requires reciprocal molecular communications between neurogenesis and angiogenesis in the nervous system (Walchli et al., 2015). The nervous and vascular networks develop in a similar stereotypic pattern and navigate nearby toward their targets as needed (Carmeliet and Tessier-Lavigne, 2005). Besides anatomical similarities, during embryonic development, these two systems share familiar guiding cues and signaling pathways to establish accurate nerve-vessel coordination, such as slits and the Roundabout receptors (Guijarro-Munoz et al., 2012), netrins and UNC5B (Castets and Mehlen, 2010), semaphorins and neuropilins, ephrins and ephr receptors, Wnt and Notch signaling pathways (Adams and Eichmann, 2010; Thomas et al., 2013). This mutual interdependency is remarkably conserved among vertebrates, including zebrafish (Melani and Weinstein, 2010). During embryonic neurogenesis and post-injury restoration, angiogenic factors can also act as neurotrophic factors to regulate cell proliferation, differentiation, and neuronal remodeling in stem cell niches (Hatakeyama et al., 2020). For instance, vascular endothelial growth factors (VEGFs) via Notch signaling pathways are essential in promoting angiogenesis, vascular formation, proliferation, and neurogenesis in adult stem cells (Lin et al., 2019). Likewise, angiopoietins, including ANGPT1, ANGPT2, and ANGPT3, are vital for cardiovascular development and adult vessel regeneration. ANGPT1 primarily binds to its endothelial receptor tyrosine kinase (TEK) and activates the downstream PI3K/AKT and Rho family GTPase to support vessel development and stabilize the vasculature (Gale and Yancopoulos, 1999; Koh, 2013). Growing evidence indicates that ANGPT1 can induce neuronal differentiation and neurite outgrowth in *ex vivo* systems (Bai et al., 2009; Chen X. et al., 2009; Rosa et al., 2010), apart from its prominent role in angiogenesis. Angpt1, acting as a neurotrophic factor, prevents blood-brain barrier (BBB) leakage in ischemic rats through increased vascular density and neuronal differentiation (Meng et al., 2014). Previous studies have suggested that the angpt1/tek pathway is not crucial in zebrafish compared to its essential function in mammals (Gjini et al., 2011). Nevertheless, angpt1 appears to be a necessary regulator of brain size in guppies and zebrafish (Chen et al., 2015), and notch1 is crucial for this regulation.

This study explored the possibility that angpt1 is vital in brain development. Instead of interacting with *tek*, *angpt1* could use another signaling system to regulate neuronal development in the zebrafish brain. Previous studies have shown that fiber outgrowth induced by Angpt1 in neuronal PC12 cells is independent of Tek and dependent on beta 1 integrin (Chen X. et al., 2009), and cell adhesion, not limited to endothelial cells, Angpt1 is not reliant on Tek but requires integrins (Carlson et al., 2001).

To investigate the function of *angpt1* and its interaction with *tek* and *integrin, beta 1b* (*itgb1b*) in neurogenesis, we utilized loss-of-function and gain-of-function approaches in zebrafish. We first examined the overt vasculature and neural phenotype of the *angpt1*^*sa*14264^ mutant zebrafish. Along with the *angpt1*^*sa*14264^ mutant, we further analyzed the neural development in the *itgb1b*^*mi*371^ and *tek*^*hu*1667^ mutant lines and found similar neural defects in the *itgb1b*^*mi*371^ mutant line. We overexpressed zebrafish *angpt1* driven by ubiquitous and neural-specific promoters to reveal its neurogenic effects in the zebrafish larval brain. Altogether, our findings suggest that *angpt1* and *itgb1b* have vital dual functions in embryonic neurogenesis via notch and wnt pathways in a *tek*-independent manner in the zebrafish brain.

## 2 Materials and methods

### 2.1 Zebrafish strain and maintenance

The zebrafish Turku wild-type (WT) strain was obtained from our breeding line maintained in the laboratory for over two decades (Kaslin and Panula, 2001; Chen et al., 2020). The fish were raised at 28°C and staged in hours post-fertilization (hpf) or days post-fertilization (dpf) as described previously (Kimmel et al., 1995). The *angpt1* mutant allele was generated by the Sanger Centre Zebrafish Project (The Wellcome Trust Sanger Institute, Hinxton, Cambridge, UK). The F3 heterozygous *angpt1*^*sa*14264/+(*TL*)^ strain was obtained from the European Zebrafish Resource Center and outcrossed with the Turku WT strain at least twice in our laboratory. All experiments used F6 or F7 progeny from in-crossed F5 or F6 *angpt1*^*sa*14264/+^ parents. The *itgb1b*^*mi*371^ mutant line (Iida et al., 2018) done by an N-ethyl-N-nitrosourea mutagenesis screen was provided by the National BioResource Project Zebrafish (NBRP).^1^ The *tek*^*hu*1667^ mutant line was kindly provided by Dr. Stefan Schulte-Merker (Gjini et al., 2011). The experimental procedures in this study were conducted under the Office of the Regional Government of Southern Finland’s permits, in agreement with the European Convention’s ethical guidelines.

### 2.2 Fin clipping and genomic DNA extraction of larval and adult zebrafish

Individual tail clippings were incubated in 50 μl of lysis buffer (10 mM Tris-HCl pH8.3, 50 mM KCl, 0.3% Tween 20 and 0.3% NP40) at 98°C for 10 min and then transferred to the ice for 2 min followed by 1 μl of Proteinase K (20 mg/ml) digestion to remove proteins and then incubated at 55°C for at least 4 h to prepare genomic DNA for lysis. The samples were set at 98°C for 10 min and quenched on ice to inactivate Proteinase K. The high-resolution melting (HRM) curve acquisition and analysis were used to detect the point mutation. Primers flanking the mutation site were designed using Primer-BLAST,^2^ and primer sequences were listed in Table 1. The HRM analysis was done on the LightCycler 480 instrument (Roche), and reaction mixtures were: 1X LightCycler 480 HRM master mix (Roche), 2 mM MgCl2, and 0.15 μM primer mixtures. The PCR cycling protocol was: one cycle of 95°C for 10 min; 45 cycles of 95°C for 10 s, 60°C for 15 s, and 72°C for 20 s, followed by Melting curve acquisition: one cycle of 95°C for 60 s and 40°C for 60 s. PCR products were denatured at 95°C for 60 s, renatured at 40°C for 60 s, and melted at 60°C to 95°C with 25 signal acquisitions per degree. Melting curves were generated over a 65–95°C range. Curves were analyzed using the LightCycler 480 gene-scanning software (Roche) (Chen et al., 2020). The deviations of the curves indicating sequence polymorphism were identified by the Gene Scanning program (Roche) as described previously (Chen et al., 2020). Those showing similar melting curves were characterized as the same genotype, and the point mutation was verified by Sanger sequencing. We genotyped all embryos of *angpt1*^*sa*14264^, *itgb1b*^*mi*371^, and *tek*^*hu*1667^ mutants used in this study.

**TABLE 1:** List of primers used in this study.

Gene	5′-Forward primer	5′-Reverse primer	Type	Accession number
*actb1*	CGAGCAGGAGATGGGAACC	CAACGGAAACGCTCATTGC	qPCR	NM_131031.1
*angpt1*	CGTCGCGGTTGGAAATTCAG	AGGTCAGATTTCTCCGTCCG	qPCR	NM_131813.1
*angpt2a*	TGATGCAGGACAACCCAGAT	ATCCACCTCCATCTGTCTCCA	qPCR	NM_001278825.2
*angpt2b*	TGTGCGATCAGAGATGGAGC	GGGAGACCTCCTGAGTTTGC	qPCR	NM_131814.1
*apoea*	GAACAAAGCTAACTCCTATGCCA	GTGAAGCACGAGACTGAACC	qPCR	NM_001020565
*apoeb*	AACGCCTGAACAAGGACACA	GTATGGCTGGAAACGGTCCT	qPCR	NM_131098.1
*epha4a*	TTCACCGCTGCTGGCTATAC	TCTGGGACAACATTCCCTGC	qPCR	XM_005171316.4
*ephb4a*	TCGGACATTCGCCGGATATG	CCAGTAACAAGCAGCGGAGA	qPCR	NM_131414.1
*ephb4b*	GAGCACTCGACTCCTTCCTG	TCATTCCAGACGCGATTCCA	qPCR	NM_131417.1
*figf/vegfd*	GAGCCTGTGCTGGTGAAAAT	CTGCCATCCAGTCCCATATT	qPCR	NM_001040178.1
*gfap*	GAAGCAGGAGGCCAATGACTATC	GGACTCATTAGACCCACGGAGAG	qPCR	NM_131373.2
*itga5*	TGTACGAGATTCAGGGCACG	ACAGTCCACACTGGAGCAAG	qPCR	NM_001004288.2
*itgav*	ACTGTCTCAAGGCTAGTGGC	GAACAGCACCCGTTTGATGG	qPCR	NM_001033721.1
*itgb1a*	AGCTTCATCTGCGAATGCGA	CGACCCACACGACCCTTATT	qPCR	NM_001034971.1
*itgb1b*	TACGTCTCCCACTGCAAGAAC	ACAGCCTTTTGCGGTGATCG	qPCR	NM_001034987.1
*nes*	GACAGCCTGATGTTGGATAGACAA	CTGTCCAGCAAAGCTCTGTATGTT	qPCR	XM_001919887.7
*notch1a*	AGAGCCGGATTCAGCGGTC	TTACAGGGACGTGGAGAACAAG	qPCR	NM_131441.1
*pax2a*	CCGCGTTATTAAGTTCCCCT	TGGCGTATCCATCTTCAATCC	qPCR	NM_131184.2
*pax2b*	TACCTGGATATCCACCTCAC	GAGCCTCTGGATGCACTATA	qPCR	NM_131640.1
*pax5*	GTGGCAGTGACGCAGGTTTCT	GCTGTTCTTCATCTCCTCCAA	qPCR	NM_131638.1
*pcna*	ACGCCTTGGCACTGGTCTT	CTCTGGAATGCCAAGCTGCT	qPCR	NM_131404.2
*sox2*	CCTATTCGCAGCAAAGCACG	GGAATGAGACGACGACGTGA	qPCR	NM_213118.1
*sox10*	AGGGTCACCATTGGGTGATG	TCGCCTGATTTTCCTCCCTG	qPCR	NM_131875.1
*tek*	TCAACACAGAGCCCTACAGC	GTCCAGTCGCACCAGCG	qPCR	NM_131461.1
*th*	GACGGAAGATGATCGGAGACA	CCGCCATGTTCCGATTTCT	qPCR	NM_131149.1
*th2*	CTCCAGAAGAGAATGCCACATG	ACGTTCACTCTCCAGCTGAGTG	qPCR	NM_001001829.1
*tie1*	GGCTCTTCTGGCCCTCTTTT	TTGGTCGTCGGGTAAGTGTG	qPCR	NM_001346150.1
*vegfaa*	AAAAGAGTGCGTGCAAGACC	GACGTTTCGTGTCTCTGTCG	qPCR	NM_131408.3
*vegfab*	TGTTGGTGGAAATTCAGCAG	CACCCTGATGACGAAGAGGT	qPCR	NM_001328597.1
*vegfba*	TGGGAGACGAATCACCTCTT	GCTGCACAAGTTCATGCTTC	qPCR	XM_021481111.1
*vegfc*	CGACAGCAGCACTCAATCAT	CTGACACGTCTCCTCATCCA	qPCR	NM_205734.1
*wnt1*	ACGCTATCTGACCAACCTGC	GGATCCAGACATCCCGTGAC	qPCR	NM_001201398.1
*wnt2bb*	GTGGCGCTAAGGAGTGGATT	TATACGAACGCTGCCTCACG	qPCR	NM_001044344.1
*wnt10b*	CTCGTGATATACACGCTCGCA	CCATGGCACTTGCACTTTCTC	qPCR	NM_178219.2
*angpt1*	AGAGCTACCGGAAACAGCAC	GCGTCTTTAGCACAGAGGCT	HRM	NC_007130.7
*itgb1b*	CTGCGTTTGTGGAACGTGCG	GTACCTCCACAGAGCTTGTTGTT	HRM	CABZ01058720
*tek*	GGGAGAGAACTTTGTGGCGA	GTGCAGACTCTCGCCTGATC	HRM	CU855580

### 2.3 Construction of transgenic *angpt1* expression plasmids

Zebrafish *angpt1* plasmids for ectopic expression were constructed by Gateway cloning and Tol2kit based on Kwan et al. (2007). 5′-entry vectors, including pENTR5′-*ubi* (ubiquitin promoter) (plasmid #27230), p5E-*elavl3* (plasmid #75025), and p5E-*gfap* (plasmid #75024), were obtained from Addgene (Mosimann et al., 2011; Don et al., 2017), and p5E-*h2afx* was from the Tol2Kit. The intention of using various promoters was to investigate the effects of the ubiquitous, neuronal, and non-neuronal expression of *angpt1* on neurogenesis. The full-length coding sequence of zebrafish *angpt1* (NM_131813) was verified and reported by Chen et al. (2015). The open reading frame *angpt1* was added to *Hin*dIII and *Eco*RI sites and ligated to the middle-entry pME-MCS vector. Gateway entry vectors (pENTR5′-promoter, pME-*angpt1*, and p3E-EGFPpA) and a destination vector pDestTol2CG2 were assembled using Gateway LR clonase II Mix (ThermoFisher, 11791-020). Transgene expression in zebrafish was generated using Tol2-mediated transgenesis. The Tol2 pCS-TP plasmid kindly provided by Dr. Koichi Kawakami (Kawakami et al., 2004) was used to synthesize mRNA encoding Tol2 transposase using the SP6 mMESSAGE mMACHINE kit (Ambion, Austin, TX, USA). 20 ng/μl of ectopic *angpt1* expression plasmid and 50 ng/μl of *Tol2* mRNA were injected into one-cell stage embryos, and the injection volume was two nl. The control group was injected with 20 ng/μl of the destination vector pDestTol2CG2 with 50 ng/μl *Tol2* mRNA. Injected embryos with GFP fluorescence in the heart, showing successful genomic integration, were collected for further experiments.

### 2.4 RNA isolation and cDNA synthesis

For quantitative real-time PCR analysis, total RNA was extracted from 30 pooled embryos or ten pooled genotyped larvae for each sample using the RNeasy mini Kit (Qiagen, Valencia, CA, USA). One or two μg of total RNA was reverse transcribed using the SuperScriptTM III reverse transcriptase (Invitrogen, Carlsbad, CA, USA) to synthesize cDNA according to instructions provided by the manufacturer.

### 2.5 Quantitative real-time PCR (qPCR)

qPCR was performed using the Lightcycler^®^ 480 SYBR Green I Master (Roche) in the LightCycler 480 instrument (Roche, Mannheim, Germany). Primers (Table 1) for amplification were designed by Primer-BLAST (NCBI). For developmental qPCR, β-*actin* and *lsm12b* were used as reference controls; for the 3-dpf qPCR analysis, β-*actin* and *ribosomal protein L13a* (*rpl13a*) were used to standardize the results (Hu et al., 2016). The melting curve analysis confirmed all primer sets’ specificity to amplify only a single product of the correct size. No peaks appeared when RNA samples without reverse transcriptase were added. Cycling parameters were as follows: 95°C for 5 min and 45 cycles of the following, 95°C for 10 s, 60°C for 15 s, and 72°C for 20 s. Fluorescence changes were monitored after each cycle. Dissociation curve analysis was performed (0.1°C per increase from 60°C to 95°C with continuous fluorescence readings) at the end of the cycles to ensure that only a single amplicon was obtained. All reactions were performed in technical duplicates, and data are shown in biological triplicates. Results were analyzed with the LightCycler 480 software with the default settings. C_T_ values of the genes of interest were normalized to the internal reference genes (Hu et al., 2016). The relative quantitative gene expressions were calculated by the comparative C_T_ method using the formula (2^–ΔCT^) to reveal the actual different expression levels of all target genes instead of presenting fold changes (2^–ΔΔCT^) (Schmittgen and Livak, 2008). Since the gene expression changes showed similar trends when normalized to different housekeeping genes, including *elfa*, *rpl13a*, β-*actin*, or *lsm12b* (data not shown), the results normalized to β-*actin* or *lsm12b* are shown in this study.

### 2.6 Fixation of embryos

Turku WT embryos were treated with 0.03% 1-phenyl-2-thiourea (PTU) in E3 medium (5 mM NaCl, 0.17 mM KCl, 0.4 mM CaCl2, and 0.16 mM MgSO4) from 13 hpf to 3 dpf for preventing pigmentation and the PTU-treated embryos were fixed in 4% paraformaldehyde (PFA) in PBS overnight at 4°C. The *angpt1*^*sa*14264^ and *itgb1b*^*mi*371^ embryos were sensitive to PTU treatment, so the mutant embryos were fixed in 4% PFA in PBS for 3 h and then treated with 3% H_2_O_2_/0.5% KOH in PBS for 30 min at 25°C (Thisse and Thisse, 2008) and washed several times with PBS to generate transparent samples for *in situ* hybridization. The fixed embryos were dehydrated with a graded methanol series (25, 50, and 75% for 10 min each) and stored at −20°C in 100% methanol. Samples for Immunostaining were fixed in 2% PFA or 4% 1-ethyl-3 (3-dimethylamino propyl)-carbodiimide (EDAC, Carbosynth, Berkshire, UK). The fixed brains were dissected to enhance antigen presentation and improve image quality.

### 2.7 Whole-mount double fluorescent *in situ* hybridization (WISH)

As described previously, whole-mount *in situ* hybridization (WISH) was performed on 4% PFA fixed embryos (Chen Y. C. et al., 2009). Briefly, antisense and sense digoxigenin (DIG) UTP-labeled or fluorescein (FLUO)-UTP-labeled probes were generated using the DIG RNA labeling kit (Roche Diagnostics, Germany), following the manufacturer’s instructions. The WISH followed Thisse’s protocol (Thisse and Thisse, 2008). The specificity of antisense riboprobes was determined by using sense probes showing faint or no staining signals. The pre-hybridization and hybridization were conducted at 65°C. Colorimetric *In situ* hybridization was conducted with sheep anti-digoxigenin-AP Fab fragments (1:10,000; Roche Diagnostics, Germany) conjugated with alkaline phosphatase. The colorimetric staining was carried out with chromogen substrates (nitro blue tetrazolium and 5-bromo-4-chloro-3-indolil-phosphate). Double-fluorescent WISH was conducted according to the previous description (Chen et al., 2016; Hauptmann et al., 2016), and RNA probes were incorporated with DIG-UTP or FLUO-UTP. Three sets of double WISH were performed on 2-dpf fixed fish, which were co-hybridized with antisense DIG-UTP labeled *angpt1* and antisense FLUO-UTP labeled *notch1a* probes, antisense DIG-UTP labeled *angpt1* and antisense FLUO-UTP labeled *delta* (*dla*) probes, or antisense DIG-UTP labeled *angpt1* and antisense FLUO-UTP labeled *nestin (nes)* probes. Hybridized probes labeled with DIG-UTP mix were reacted with anti-DIG-POD antibody (1:500; Roche Diagnostics GmbH, Germany), followed by the detection with Alexa Fluor 594 Tyramide reagent (B40915, Invitrogen, Eugene, OR); hybridized probes labeled with FLUO-UTP mix were reacted with anti-FLUO-POD antibody (1:250; Roche Diagnostics GmbH, Germany), followed by the detection with Alexa Fluor 488 Tyramide reagent (B40912, Invitrogen, Eugene, OR, USA). The sense probes labeled with DIG-UTP and FLUO-UTP mix were utilized as a negative control, where no fluorescence was detected in the stained samples.

### 2.8 EdU labeling and double immunocytochemistry

The Click-iT™EdU Alexa Fluor 488 imaging kit (Molecular Probes) was used according to the manufacturer’s instructions with minor modifications to detect the S-phase proliferation of dividing cells. Briefly, 3-dp or 5-dpf larvae were incubated in 0.5 mM EdU/E3 buffer with 1% DMSO for 24 h at 28°C. EdU-labeled samples were transferred back to E3 for 30 min and fixed in 2% PFA overnight at 4°C with agitations (Chen et al., 2020). The fixed specimens were dissected to enhance sample penetration. Dissected brains were incubated with monoclonal mouse anti-HuC/D (1:500, Invitrogen, Cat.No: A21271), anti-tyrosine hydroxylase (TH1) monoclonal mouse antibody (1:1,000; Product No 22941, Immunostar, Hudson, WI, USA), anti-GABA 1H [1:1,000; (Karhunen et al., 1993; Kukko-Lukjanov and Panula, 2003)] and mouse anti-zrf1 (Gfap; 1:1,000, Zebrafish International Resource Center). The specificities of the anti-GABA, anti-histamine antisera, and commercial anti-mouse monoclonal TH antibodies have been previously verified (Kaslin and Panula, 2001). The following secondary antibodies were applied: Alexa Fluor^®^ 488 and 647 anti-mouse or anti-rabbit IgG (1:1,000; Invitrogen, Eugene, OR, USA). After immunostaining, labeled specimens were fixed in 4% PFA for 20 min at RT and then Incubated in a 1XClick-iT EdU cocktail containing Alexa 568-azide for 1 h in the dark at room temperature. After removal of the reaction cocktail and rinsing 3 times in 1XPBST for 10 min, the samples were mounted in 80% glycerol/PBS for confocal microscopy.

### 2.9 Retrograde labeling

Reticulospinal neurons were visualized by retrograde labeling with the fluorescent dye Dextran Alexa Flour 488 10,000 KDa (Invitrogen D22910) applied to the spinal cord of 3-dpf larvae (Alexandre et al., 1996). Briefly, anesthetized larvae were placed on a 1% agarose template. The labeling dye was applied on a spinal cord lesion site between smites 12 and 15 made with a 30G needle in the dark for 2 min, and injected embryos were incubated in an E3 medium for 2 h. The labeled embryos were euthanized on ice and fixed in 4% PFA. The fixed specimens were bleached (0.8% KOH, 0.9% H_2_O_2_, and 0.1% Tween-20) to avoid pigment disturbance while imaging and mounted in 80% glycerol for confocal microscopy.

### 2.10 Imaging

Bright-field images were taken with a Leica DM IL inverted microscope. A Leica DM IRB inverted microscope with DFC 480 charge-coupled device camera was used to collect z-stacks of photographs processed with Leica Application Suite software. Immunofluorescence samples were examined using a Leica TCS SP2 AOBS confocal microscope. For excitation, an Argon laser (488 nm), green diode laser (561 nm), and red HeNe laser (633 nm) were used. Emission was detected at 500−550 nm, 560−620 nm, and 630−680 nm, respectively. Crosstalk between the channels and background noise was eliminated with sequential scanning and frame averaging, as described earlier (Sallinen et al., 2009). Stacks of images taken at 0.2–1.2 μm intervals were compiled, and the maximum intensity projection algorithm was used to produce final images with Leica Confocal software and Imaris imaging software version 6.0 (Bitplane AG, Zurich, Switzerland).

### 2.11 Cell quantification

Numbers of Edu-positive, GABA-positive, histamine-positive, HuC-positive, and TH-positive cells were counted with 1-μm steps throughout the entire Z-stack images using the Multipoint tool in Fiji software (Sallinen et al., 2009; Schindelin et al., 2012). Cell counting was conducted in a blinded experiment.

### 2.12 Experimental design and statistical analyses

Data analysis utilized GraphPad Prism v.7.0c software (San Diego, CA, USA). Sample sizes and statistical methods are stated individually in the figure legends. Kruskal–Wallis test with Dunn’s multiple comparisons test was utilized for the sample number (n) below 5. In qPCR experiments, three independent biological replicates having three genotypes with 30 pooled embryos per genotype (number of values is 9 with 270 embryos) were utilized to the minimum amount of animal resources to meet the requirement of a power dynamic. When the sample number is above 5, *P*-values were generated by an ordinary one-way analysis of variance (ANOVA), an ordinary two-way ANOVA for multiple comparisons, and a Student’s *t*-test (unpaired test) to compare two groups. Data were presented as mean ± SD. *P*-value < 0.05 was considered statistically significant.

## 3 Results

### 3.1 Zebrafish *angpt1* mRNA detected in the neurogenic domain of the 2-dpf brain

**FIGURE 1:**
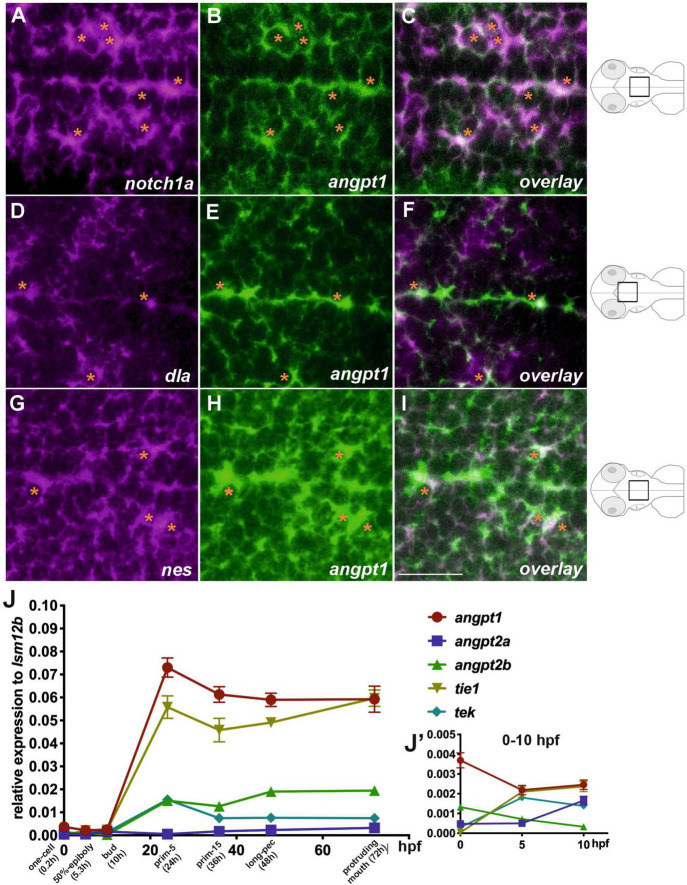
Co-localization of angpt1 with neurogenic markers in midline proliferative zone (shown schematically on the right). Double FISH in 2-dpf brain on 1.0 um thick optical sections using antisense RNA probes simultaneously hybridized against *notch1a* **(A)** and *angpt1* **(B)** and the overlay pattern of **(A,B)** is shown in **(C)**. Double FISH in the 2-dpf brain using antisense RNA probes against *dla* **(D)** and *angpt1* **(E)**, and the overlay pattern of **(D,E)** is shown in **(F)**. Double FISH in 2-dpf brain using antisense RNA probes against *nes* **(G)** and *angpt1* **(H)**, and the overlay pattern of **(G,H)** is shown in **(I)**. Dynamic expression of angiogenic factors through the one-cell stage to 72 hpf by qPCR is shown in **(J)**. *P* = 0.0428. **(J’)** Expression of angiogenic factors from 0 hpf to 10 hpf. Images are stacks of merged Z-slices. Tangerine stars indicate *angpt1*-positive cells co-localized with *notch1a*, *dla*, and *nes*-expressing cells. Statistical analysis of the qPCR result is shown in mean ± SD (*N* = 3 groups per stage, 20–30 embryos in one group) by Kruskal–Wallis test with Dunn’s multiple comparisons test. Scale bar is 20 μm.

We first studied the spatiotemporal distribution of angiogenic factors during zebrafish embryogenesis. We conducted extensive characterization of expression patterns of *angpt1*, *angpt2a*, *angpt2b*, *tie1*, and *tek* (*tie2*) from the zygotic period (0 hpf) to the hatching period (72 hpf) (Supplementary Figures 1A–G). The WISH results showed that angiopoietins and their receptors were ubiquitously expressed in the ectoderm and mesoderm from the blastula to the gastrula period, of which *angpt1* and *tek* displayed the most intense signals at the early segmentation period (bud) (Supplementary Figure 1C). Zebrafish neurulation starts at the 80% epiboly (end of gastrulation) stage (Lowery and Sive, 2004). We used double fluorescent *in situ* hybridization in 2-dpf zebrafish brains to gain insight into the roles of zebrafish *angpt1* in early embryonic neurogenesis. Notch1 and Notch2 and its ligands, such as Delta, are recognized as neurogenic genes (Engler et al., 2018). The Notch signaling pathway is crucial for determining neural cell fate and differentiation of neuronal cells (Artavanis-Tsakonas et al., 1999; Mueller and Wullimann, 2003). The *FISH* results showed that *angpt1* mRNA was found mainly in the *notch1a*-expressing domain (Figures 1A–C). The *angpt1* expression also co-localized with the *delta (dla)*-expressing (Figures 1D–F) and *nestin (nes)*-expressing cells (a marker for neural precursors) (Figures 1G–I) along the ventricle in the medulla oblongata, which were recognized as proliferative zones (Mahler and Driever, 2007), suggesting the neurogenic role of angpt1 in early neurogenesis. We further utilized qPCR analysis to illustrate distinctly differential expression profiles of angiopoietin factors. The *angpt1* and *tie1* expressions were more abundant than those of *angpt2a*, *angpt2b*, or *tek* during development (Figure 1J). The *angpt1* displayed the relatively highest maternal RNA expression compared with other angiogenic factors (Figure 1J’), and a maternal-to-zygotic transition change appeared at five hpf, indicating its potent role during embryogenesis.

### 3.2 Dynamic changes in the expression of angiogenic factors in *angpt1*^–/–^ embryos

To investigate the biological functions of angpt1 during embryonic neurogenesis, we carried out loss-of-function experiments using the *angpt1*^*sa*14246^ mutant line generated by the TILLING (Targeting Induced Local Lesions IN Genomes) method. The *angpt1*^*sa*14246^ allele contains a C > T substitution. It introduced a premature stop codon at Q261, resulting in a truncated protein missing the entire fibrinogen-C-terminal domain, the region in which Angpt1 binds to the Tek receptor (Supplementary Figure 2A). The cerebrovascular phenotypic defects of *angpt1*^–/–^ larvae were apparent from 2 dpf (Supplementary Figures 2B–E and Supplementary Figures 3A–C) and became progressively more severe afterward. The *angpt1*^–/–^ larvae displayed smaller body length, brain, and eye sizes than their *angpt1*^+/+^ siblings (Supplementary Figures 2F–H). The *angpt1*^–/–^ larvae died within 7dpf.

**FIGURE 2:**
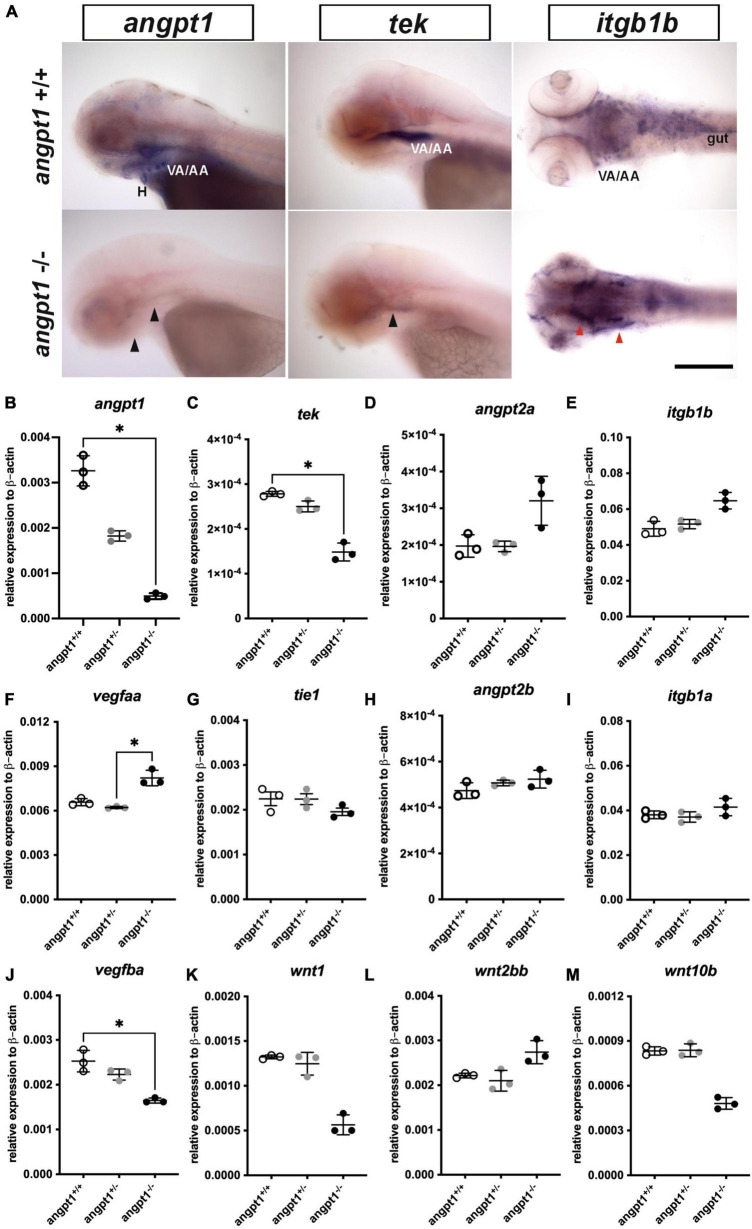
mRNA expression levels of angiogenic factors in 3-dpf *angpt1*^–/–^ larvae. **(A)** Expression patterns of *angpt1*, *tek*, and *itgb1b* in 3-pf *angpt1*^+/+^ and angpt1^–/–^ larvae done by WISH. Quantification of mRNA levels done by qPCR using 3-dpf *angpt1*^+/+^, *angpt1*^±^, and *angpt1*^–/–^ larvae shown in **(B)** *angpt1*; **(C)** *tek*; **(D)** *angpt2a*; **(E)** *itgb1b*; **(F)** *vegfaa*; **(G)** *tie 1*; **(H)** *angpt2b*; **(I)** *itgb1a* F; **(J)** *vegfba* **(K)** *wnt1*; **(L)** *wnt2bb*; **(M)** *wnt10b* level. Samples were genotyped with HRM analysis. *N* = 6 per group for WISH. *N* = 3 Replications for qPCR in each genotyped group (10-pooled embryos in one replication). H, heart; VA/AA, ventral aorta/branchial arch. Arrows indicate regions showing differential expression patterns between *angpt1*^+/+^ and *angpt1*^–/–^ larvae, the black one indicating downregulation and the red one showing upregulation. Statistical analysis of qPCR results is shown in mean ± SD by Kruskal–Wallis test with Dunn’s multiple comparisons test. *p < 0.05. Scale bar is 200 μm.

Using 3-dpf genotyped embryos, the WISH result showed that the *angpt1* and *tek* expressions were noticeably reduced in *angpt1*^–/–^ embryos (Figure 2A). In contrast, the *igtb1b* (the integrin family as a potential critical receptor of angpt1) signaling increased in *angpt1^–/–^* larvae compared with *angpt1*^+/+^ siblings (Figure 2A). Next, we used qPCR to quantify the mRNA expression levels of relevant angiogenic factors in 3-dpf *angpt1* mutant embryos. The statistical significance is stated in Table 2. Compared with *angpt1*^+/+^ siblings, the *angpt1*^±^ and *angpt1*^–/–^ larvae had about 55.8 and 15% of remaining *angpt1* mRNA detected, respectively (Figure 2B), confirming that the truncated form of the *angpt1* transcript was targeted for the nonsense-mediated decay surveillance pathway (NMD). Concomitantly with the *angpt1* downregulation, a significant decrease of *tek* expression (specific binding receptor of angpt1) was found in the *angpt1*^–/–^ embryos (Figure 2C), but *tie1* expression showed no significant differences between *angpt1*^±^ and *angpt1*^–/–^ larvae (Figure 2G). In contrast, *angpt2a* (a context-dependent antagonist of *angpt1*) (Figure 2D) and *itgb1b* (Figure 2E) were upregulated in the *angpt1*^–/–^ larvae, and this upregulation phenotype was not found in their paralogous genes, *angpt2b* (Figure 2H) and *itgb1a* (Figure 2I), respectively. Intriguingly, the expression level of *vegfaa* (Figure 2F) and *vegfba* (Figure 2J) (vascular endothelial growth factor, an angiogenic protein with neurotrophic and neuroprotective effects), were upregulated and downregulated, respectively, in the *angpt1*^–/–^ larvae. Moreover, the mRNA expression of *wnt1* (Figure 2K), *wnt2bb* (Figure 2L), and *wnt10b* (Figure 2M) (wnt family, regulators of cell fate and patterning during neurogenesis, angiogenesis, and maintaining neurovascular functions) (Menet et al., 2020) were dramatically altered in the *angpt1*^–/–^ larvae. These dynamic alterations of angiogenic factors and wnt ligands due to loss of *angpt1* may reflect angpt1 commending the effort to stabilize neurovascular formation and angiogenesis during embryonic development.

**TABLE 2:** Summary of RT-qPCR results of 3-dpf *angpt1*^–/–^ embryos.

Biological process	Gene	*angpt1* ^+/+^		*angpt1* ^±^		*angpt1* ^–/–^			*angpt1*^–/–^ vs. *angpt1*^+/+^
		Mean	SD	Mean	SD	Mean	SD	*p*-value	
Angiogenesis	*angpt1*	3.26E-03	3.33E-04	1.82E-03	1.13E-04	4.94E-04	6.77E-05	*P* = 0.0036	down*
	*angpt2a*	1.97E-04	3.05E-05	1.96E-04	1.43E-05	3.20E-04	6.66E-05	*P* = 0.0714	ns
	*angpt2b*	4.74E-04	3.42E-05	5.07E-04	1.22E-05	5.24E-04	3.86E-05	*P* = 0.3393	ns
	*figf*	1.03E-03	3.00E-05	1.12E-03	6.24E-05	1.40E-03	1.21E-04	*P* = 0.0036	up*
	*itga5*	5.61E-03	6.74E-04	5.11E-03	3.09E-04	4.62E-03	8.27E-04	*P* = 0.2964	ns
	*itgav*	7.35E-02	2.91E-03	7.44E-02	4.46E-03	7.97E-02	2.23E-03	*P* = 0.1321	ns
	*itgb1a*	3.80E-02	1.73E-03	3.71E-02	2.33E-03	4.15E-02	3.91E-03	*P* = 0.2964	ns
	*itgb1b*	4.90E-02	4.16E-03	5.16E-02	2.61E-03	6.47E-02	4.63E-03	*P* = 0.0500	up*
	*tek*	2.78E-04	5.50E-06	2.50E-04	1.22E-05	1.48E-04	2.00E-05	*P* = 0.0036	down*
	*tie1*	2.25E-03	2.66E-04	2.24E-03	2.11E-04	1.96E-03	1.43E-04	*P* = 0.2321	ns
	*vegfaa*	6.57E-03	2.40E-04	6.22E-03	6.99E-05	8.21E-03	5.23E-04	*P* = 0.0036	up*
	*vegfab*	4.84E-03	5.66E-04	4.18E-03	2.04E-04	4.40E-03	2.57E-04	*P* = 0.1679	ns
	*vegfba*	2.53E-03	2.42E-04	2.23E-03	1.23E-04	1.65E-03	5.53E-05	*P* = 0.0107	down*
	*vegfc*	1.07E-03	7.12E-05	1.00E-03	1.08E-04	1.12E-03	6.56E-05	*P* = 0.2536	ns
Neural patterning	*epha4a*	1.17E-02	7.58E-04	1.13E-02	3.99E-04	1.09E-02	3.67E-04	*P* = 0.0857	ns
	*ephb4a*	9.28E-03	4.81E-04	8.26E-03	4.43E-04	8.66E-03	4.27E-04	*P* = 0.1321	ns
	*ephb4b*	5.10E-03	2.49E-04	4.77E-03	1.10E-04	5.02E-03	1.45E-04	*P* = 0.1365	ns
	*sox2*	3.14E-02	5.56E-04	2.98E-02	2.35E-03	2.05E-02	7.33E-04	*P* = 0.0500	down*
	*sox10*	3.43E-03	3.25E-04	2.98E-03	3.98E-04	2.70E-03	8.43E-04	*P* = 0.4393	ns
	*wnt1*	1.32E-03	2.10E-05	1.25E-03	1.27E-04	5.65E-04	1.11E-04	*P* = 0.0250	down*
	*wnt2bb*	2.22E-03	5.24E-05	2.10E-03	2.32E-04	2.74E-03	2.57E-04	*P* = 0.0500	up*
	*wnt10b*	8.33E-04	2.77E-05	8.38E-04	4.30E-05	4.81E-04	3.88E-05	*P* = 0.0714	ns
Neuronal and glial development	*apoea*	1.94E-01	2.61E-02	1.79E-01	1.92E-02	2.03E-01	9.53E-03	*P* = 0.0714	ns
	*apoeb*	2.71E-01	2.19E-02	2.47E-01	2.13E-02	3.32E-01	2.86E-02	*P* = 0.0250	up*
	*gfap*	3.25E-02	3.75E-03	2.97E-02	3.59E-03	3.90E-02	1.62E-03	*P* = 0.0500	ns
	*nes*	2.34E-03	2.47E-04	2.19E-03	2.58E-04	1.37E-03	2.13E-04	*P* = 0.0250	ns
	*notch1a*	9.38E-03	1.78E-03	7.71E-03	5.66E-04	4.99E-03	4.23E-04	*P* = 0.0107	down*
	*pax2a*	9.41E-03	9.27E-04	8.69E-03	1.19E-03	1.06E-02	1.37E-03	*P* = 0.3821	ns
	*pax2b*	4.13E-03	5.03E-04	3.68E-03	2.72E-04	4.62E-03	4.06E-04	*P* = 0.0857	ns
	*pax5*	3.99E-03	5.12E-04	3.67E-03	4.29E-04	1.96E-03	1.78E-04	*P* = 0.0700	ns
	*pcna*	1.17E-01	2.20E-03	9.61E-02	1.58E-03	4.55E-02	1.38E-02	*P* = 0.0219	down*
	*hdc*	1.16E-03	1.15E-04	1.05E-03	9.94E-05	1.02E-04	1.19E-04	*P* = 0.3607	ns
	*Th*	2.83E-03	1.80E-04	2.72E-03	7.05E-05	2.17E-03	1.89E-04	*P* = 0.0760	ns
	*th2*	2.87E-05	6.76E-06	2.92E-05	3.56E-06	4.05E-05	1.43E-05	*P* = 0.6991	ns

Data are mean ± SD. Kruskal–Wallis test with Dunn’s multiple comparisons test was used for statistical analysis *p < 0.05, ns: non-significant, down: downregulation in the *angpt1*^–/–^ compared with the angp1^+/+^ sibling, up: upregulation in the *angpt1*^–/–^ compared with the *angp1*^+/+^ sibling.

### 3.3 Differential expression of neurogenesis-related markers in the *angpt1*^–/–^ embryos

**FIGURE 3:**
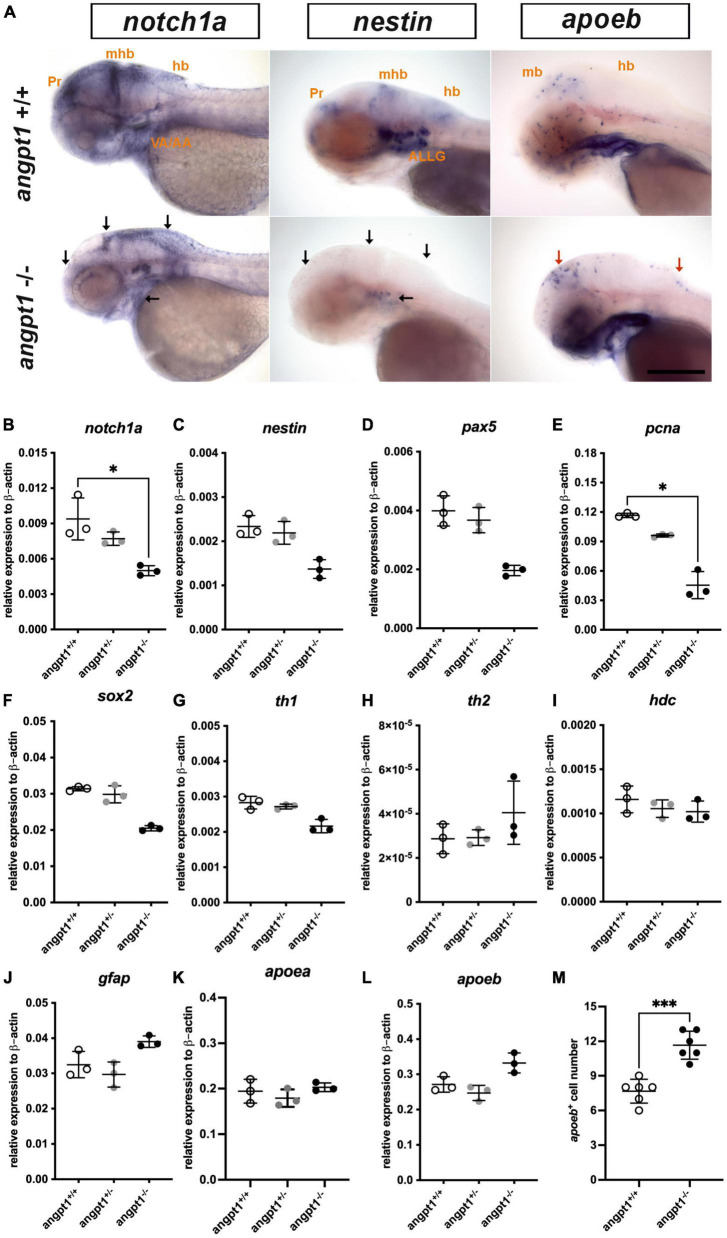
mRNA expression levels of neurogenesis markers in 3-dpf *angpt1*^–/–^ larvae. **(A)** Expression patterns of *notch1a*, *nestin (nes)*, and *apoeb* in 3-pf *angpt1*^+/+^ and *angpt1*^–/–^ larvae done by WISH. Quantification of mRNA levels done by qPCR using 3-dpf *angpt1*^+/+^, *angpt1*^±^ and *angpt1*^–/–^ larvae shown in **(B)** *notch1a*; **(C)** *nestin*; **(D)** *pax5*; **(E)** *pcna*; **(F)** *sox2*; **(G)** *th1*; **(H)** *th2*; **(I)** *hdc*; **(J)** *gfap*; **(K)** *apoea*; **(L)** *apoeb*; **(M)** Quantification of *apoeb*-positive cell numbers in the midbrain, *N* = 6, *P* = 0.0001. Samples were genotyped with HRM analysis. *N* = 6 per group for WISH. *N* = 3 For qPCR, replications in each genotyped group (10-pooled embryos in one replication). ALLG, anterior lateral line ganglion; hb, hindbrain; mhb, midbrain-hindbrain boundary; ov, otic vesicle; Pr, pretectum. Black arrows indicate regions showing decreased expression in *angpt1*^–/–^ compared with *angpt1*^+/+^ larvae. Red arrows indicate regions showing increased *angpt1*^–/–^ expression compared with *angpt1*^+/+^ larvae. Statistical analysis of qPCR results is shown in mean ± SD by the Kruskal–Wallis test with Dunn’s multiple comparisons test. Statistical analysis of *apoeb*^+^ cell numbers is shown in mean ± SD by unpaired Student *t*-test. *p < 0.05, ***p < 0.001. Scale bar is 200 μm.

Proper coordination of neurogenesis and angiogenesis is essential for developing the CNS and neurovascular system during embryonic development (Ward and Lamanna, 2004). We further investigated gene expression of relevant markers for embryonic neurogenesis in 3-dpf *angpt1*^–/–^ larvae. By WISH, we observed that early neural marker *notch1a* and *nestin* expression patterns were reduced in the head, branchial arches, and hindbrain in the *angpt1*^–/–^ larvae compared with their *angpt1*^+/+^ siblings (Figure 3A). By qPCR analysis, the mRNA levels of *notch1a* (Figure 3B), *nestin* (Figure 3C), *paired box 5* (*pax5*) (Figure 3D), *proliferating cell nuclear antigen* (*pcna*) (Figure 3E), and *SRY-box containing gene 2* (*sox2*) (Figure 3F) were lower in the *angpt1*^–/–^ larvae. The statistical significance is stated in Table 2. To determine whether specific neuron populations are affected in *angpt1*^–/–^ larvae, markers of aminergic neurons were studied. Interestingly, the expression of *tyrosine hydroxylase 1* (*th1*, a marker of dopaminergic and noradrenergic neurons) (Figure 3G) was downregulated. In contrast, the expression levels of *th2* (a marker of non-overlapping *th1* dopaminergic neurons) (Figure 3H) and *histidine decarboxylase* (*hdc*, a marker of histaminergic neurons) (Figure 3I) were unaltered in the *angpt1*^–/–^ larvae. In contrast, the mRNA expression levels of glial markers, *glial fibrillary acidic protein* (*gfap)* (Figure 3J), and *apolipoprotein Eb* (*apoeb*, Figure 3L) but not *apoea* (Figure 3K) were significantly increased in the *angpt1*^–/–^ mutants, and the number of *apoeb*-positive cells was considerably higher in the midbrain of *angpt1*^–/–^ larvae than in WT siblings (Figures 3A, M). Of neurogenic markers, genes representing proliferating and neural progenitor cells were downregulated in the 3-dpf *angpt1*^–/–^ embryos, suggesting the importance of *angpt 1* in early neurogenesis.

### 3.4 Downregulated proliferation and upregulated glial markers in 3-dpf *angpt1*^–/–^ and *itgb1b^–/–^* brains

Unlike the gross cardiovascular phenotype appearing in the *angpt1*^–/–^ mutant in this study and reported *itgb1b*^–/–^ mutant (Iida et al., 2018), the *tek*^*hu*1667^ mutant fish grow naturally without overt cardiovascular defects (Gjini et al., 2011; Jiang et al., 2020).

**FIGURE 4:**
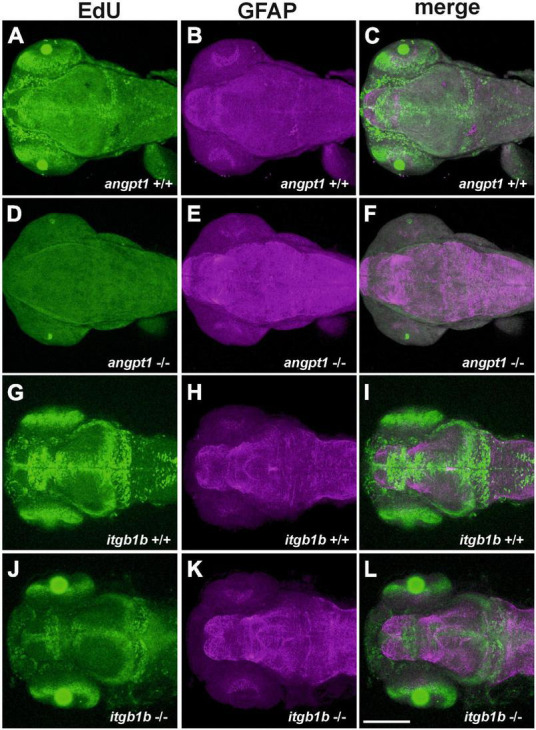
Decreased proliferation and increased gfap intensity in *angpt1*^–/–^ and *itgb1b*^–/–^ larvae. Maximum intensity projections of confocal z-stack images were made by immunostaining GFAP following the EdU proliferation assay in 3-dpf genotyped larvae. EdU labeling results shown in **(A)** *angpt1*^+/+^ **(D)** *angpt1*^–/–^ **(G)** *itgb1b*^+/+^ and **(J)** *itgb1b*^–/–^ larval brains. gfap-immunoreactivity results shown in **(B)** *angpt1*^+/+^ **(E)** *angpt1*^–/–^ **(H)** *itgb1b*^+/+^ and **(K)** *itgb1b*^–/–^ larval brains. The merge images of Edu-labeling and gfap-positive are shown in **(C)** *angpt1*^+/+^ **(F)** *angpt1*^–/–^ **(I)** *itgb1b*^+/+^ and **(L)** *itgb1b*^–/–^ larval brains. Proliferating cells are indicated in green, and gfap-positive signals are in magenta. *N* = 5 per genotyped group. Scale bars are 200 μm.

It has been reported that besides Tek, Angpt1 can bind to integrins and activate similar pathways as the angpt1-tek does (Chen X. et al., 2009), which renders it possible that the angpt1-itgb1b signaling pathway may play a more vital role than the recognized angpt1-tek pathway in the regulation of embryogenesis. To provide detailed evidence that angpt1 and itgb1b are involved in the regulation of proliferation during neurogenesis, we conducted the saturation-labeling EdU incorporated analysis followed by staining with the glial marker GFAP on 3-dpf *angpt1*^–/–^ and *itgb1b*^–/–^ larvae. A significant decrease of EdU-positive cells was found in the proliferative zones in both *angpt1*^–/–^ (Figure 4D) and *itgb1b*^–/–^ larvae (Figure 4J) compared with *angpt1*^+/+^ (Figure 4A) and *itgb1b*^+/+^ (Figure 4G) siblings, respectively. In contrast to the diminished proliferation, a substantial increase of GFAP-positive structures appeared in the forebrain and hindbrain of *angpt1*^–/–^ (Figures 4E, F) and *itgb1b*^–/–^ (Figures 4K, L) larvae compared with their WT siblings, respectively (Figures 4B, C, H, I), indicating that angpt1 and itgb1b have a considerable impact in the regulation of proliferation during embryonic neurogenesis.

### 3.5 Distortion of reticulospinal neurons and *krox20* patterns in the *angpt1*^–/–^ and *itgb1b*^–/–^ hindbrain

**FIGURE 5:**
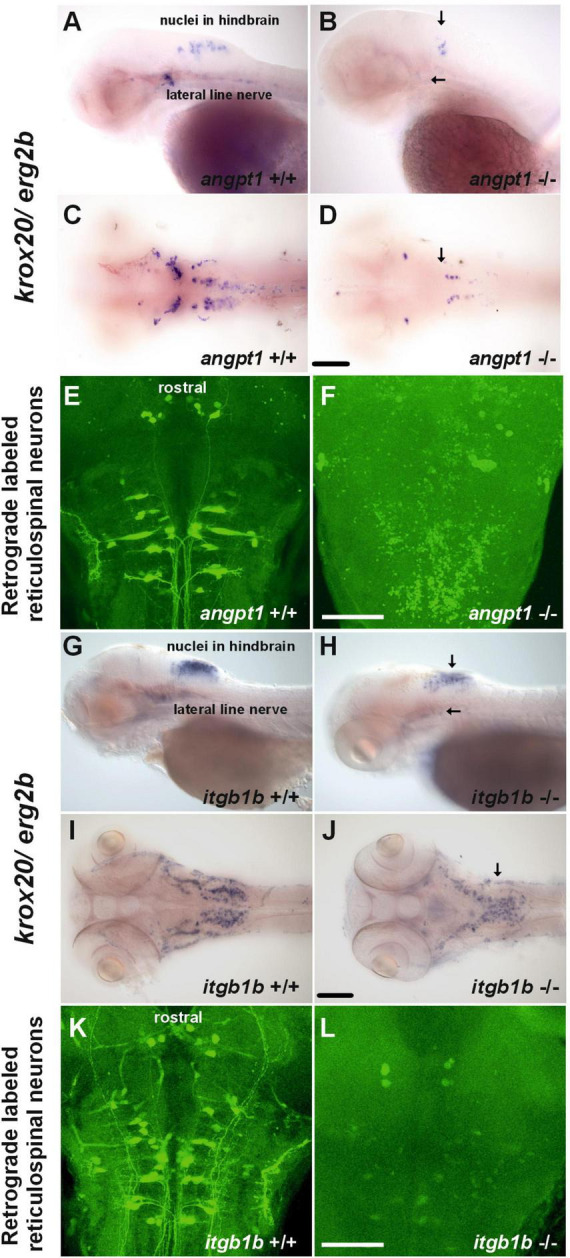
Deficiencies of reticulospinal neurons in *angpt1*^–/–^ and *itgb1b*^–/–^ larval hindbrain. Expression of *krox20* mRNA done by WISH shown in **(A)** lateral view and **(C)** dorsal view of 3-dpf *angpt1*^+/+^ and **(G)** lateral view and **(I)** dorsal view of 4-dpf *itgb1b*^+/+^ larvae. Fainter and misexpression patterns of *krox20* were found in **(B)** lateral view and **(D)** dorsal view of 3-dpf *angpt1*^–/–^ brain and in **(H)** lateral view and **(J)** dorsal view of 4-dpf *itgb1b*^–/–^ brain. Retrograde labeling of 4-dpf genotyped larvae reveals the deficiencies of reticulospinal neurons in **(F)** *angpt1*^–/–^ and **(L)** *itgb1b^–/–^* brains compared with their **(E)** *angpt1*^+/+^ and **(K)** *itgb1b*^+/+^ siblings. *N* = 5 per genotyped group in WISH and retrograde labeled analysis. Arrows indicate the abnormality of *krox20* expression patterns in the *angpt1*^–/–^ and *itgb1b*^–/–^ hindbrain and lateral line nerves. Scale bars are 200 μm.

Wnt signaling pathways are crucial for regulating rhombomere segmentation and forming the midbrain-hindbrain boundary and neuronal circuits during embryogenesis (Riley et al., 2004; Mulligan and Cheyette, 2012). As mentioned above, in the *angpt1*^–/–^ larvae, expression levels of *wnt1* (Figure 2K), *wnt2bb* (Figure 2L), and *wnt10b* (Figure 2M) were significantly affected, and these substantial alterations may cause impairments of hindbrain patterning. We further found that *krox20/egr2b*-positive cells (encoding a transcription factor playing a pivotal role in hindbrain segmentation) in the hindbrain and lateral line nerves were significantly reduced in the *angpt1*^–/–^ larvae (Figures 5B, D); similarly, fewer and disorganized *egr2b/krox20*-positive structures were found in the *itgb1b*^–/–^ larval hindbrain (Figures 5H, J) compared with their WT siblings (Figures 5A, C, G, I), respectively. Developing the hindbrain segmentation into rhombomeres orchestrates the stereotypical organization of reticulospinal, somatomotor, and branchiomotor neurons. We then performed retrograde labeling of neurons using the green fluorescent dye conjugated high-molecular-weight dextran (10,000 Mw) to investigate whether the development of reticulospinal neurons was disturbed in the *angpt1*^–/–^ and *itgb1b*^–/–^ hindbrain. Retrograde labeling results showed a severe deficiency of reticulospinal neurons in the *angpt1^–/–^* (Figure 5F) and *itgb1b*^–/–^ (Figure 5L) hindbrain, which contrasted with the normal patterning of their WT siblings (Figures 5E, K), respectively. Lack of functional *angpt1* and *itgb1b* leads to impairments of *notch1a*, *wnt* ligands, and *krox-20* expression, which may have contributed to the deficiency of the reticulospinal neurons in the larval hindbrain.

### 3.6 Deficient dopaminergic and histaminergic neurons in *angpt1* and *itgb1b* mutant brains

**FIGURE 6:**
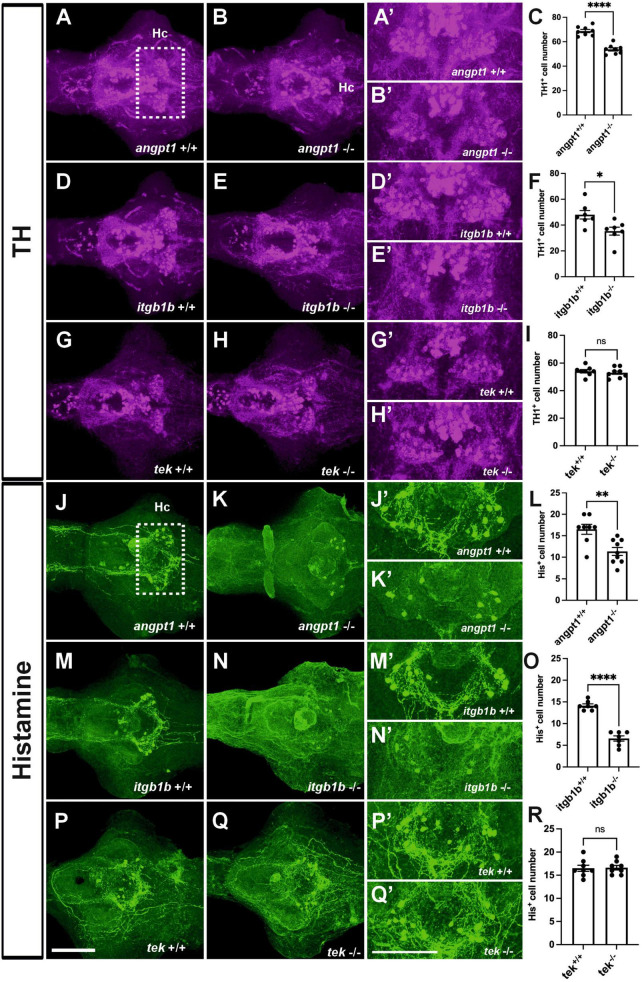
Deficient dopaminergic and histaminergic neurons are found in *angpt1*^–/–^ and *itgb1b*^–/–^ brains but normally developed in *tek*^–/–^ brains. TH1-immunostaining images of 4-dpf shown in **(A)** *angpt1*^+/+^ **(B)** *angpt1*^–/–^ **(C)** Quantification of TH1-positive cell numbers in the Hc region (*N* = 8 per group, *P* < 0.0001) **(D)** *itgb1b*^+/+^ **(E)** *itgb1b*^–/–^ **(F)** Quantification of TH1-positive cell numbers in the Hc region (*N* = 7 per group, *P* = 0.0153) **(G)** *tek*^+/+^ **(H)** *tek*^–/–^ and **(I)** Quantification of TH1-positive cell numbers in the Hc region (*N* = 8 per group, *P* = 0.5392). Histamine (His)-immunostaining images of 4-dpf show in **(J)** *angpt1*^+/+^ **(K)** *angpt1*^–/–^ **(L)** Quantification of His-positive cell numbers in the Hc region (*N* = 8–9 per group, *P* = 0.003) **(M)** *itgb1b*^+/+^ **(N)** *itgb1b*^–/–^ **(O)** Quantification of His-positive cell numbers in the Hc region (*N* = 7 per group, *P* < 0.0001) **(P)** *tek*^+/+^ **(Q)** *tek*^–/–^ and **(R)** Quantification of His-positive cell numbers in the Hc region (*N* = 8 per group, *P* = 0.8814). **(A’–Q’)** Show the high magnification images corresponding to the white rectangular area in the Hc region shown in **(A)** and an equivalent area in **(B–Q)**. TH-positive cells display in magenta, and His-positive cells display in green. Hc, caudal hypothalamus. Data represent the mean ± SD. n unpaired student *t*-test was used for statistical analysis, *p < 0.05, **p < 0.01 and ****p < 0.0001. Scale bars are 200 μm.

In the hypothalamus, a high level of neurogenesis is continuously active, and the most representative neurotransmitter systems, including dopaminergic, GABAergic, histaminergic, and serotonergic neuron groups, are located there (Kaslin and Panula, 2001; Xie and Dorsky, 2017). Hypothalamic neurogenesis and patterning depend on several morphogen signals, including Notch/Delta signaling and wnt pathways, for establishing regional identity and functional circuitry (Aujla et al., 2015; Machluf et al., 2011). When angpt1 and itgb1b signaling is lacking, which causes impairments of notch and wnt pathways, we reasoned that the development of neuron populations in the hypothalamus might be affected. We then investigated dopaminergic and histaminergic populations in the 4-dpf larval brain by quantifying the immunostained cell numbers in the caudal hypothalamus. The significant statistics is stated in the figure legend. In the *angpt1*^–/–^ and *itgb1b*^–/–^ larval brains, a significant decrease in TH1-positive cells was evident in the caudal hypothalamus (Figures 6B, B’, C, E, E’, F). Likewise, histamine-positive cells were significantly reduced in both mutants (Figures 6K, K’, L, N, N’, O). In contrast to these two mutants, the number of TH1-positive cells and histaminergic cells remained intact in *tek*^–/–^ mutants (Figures 6H, H’, I, Q, Q’, R), similar to their WT siblings, respectively (Figures 6A, A’, D, D’, G, G’, J, J’, M, M’, P, P’). The deficiency of dopaminergic and histaminergic populations in the *angpt1*^–/–^ and *itgb1b*^–/–^ brains indicates the significance of angpt1 and itgb1b for differentiation and maintenance of developing and mature neurons during brain development.

### 3.7 Neural overexpression of zebrafish *angpt1* upregulating proliferation and HuC/D positive neuronal precursor cells in zebrafish larval brains

Lack of functional angpt1 revealed downside effects on embryonic neurogenesis and angiogenesis. We considered that angpt1 overexpression might promote cell proliferation and enhance vessel formation during embryogenesis. We employed a gain-of-function system by injecting a transgene plasmid containing Tol2 transposase sites and encoded a full-length zebrafish *angpt1* driven by the various promoters to overexpress *angpt1* ubiquitously or neural specifically *in vivo* along with a heart-specific marker, the *cmlc2* promoter driving *gfp* expression. As a result, embryos with GFP expression in the heart, indicating successful transgene integration, were selected for further experiments.

**FIGURE 7:**
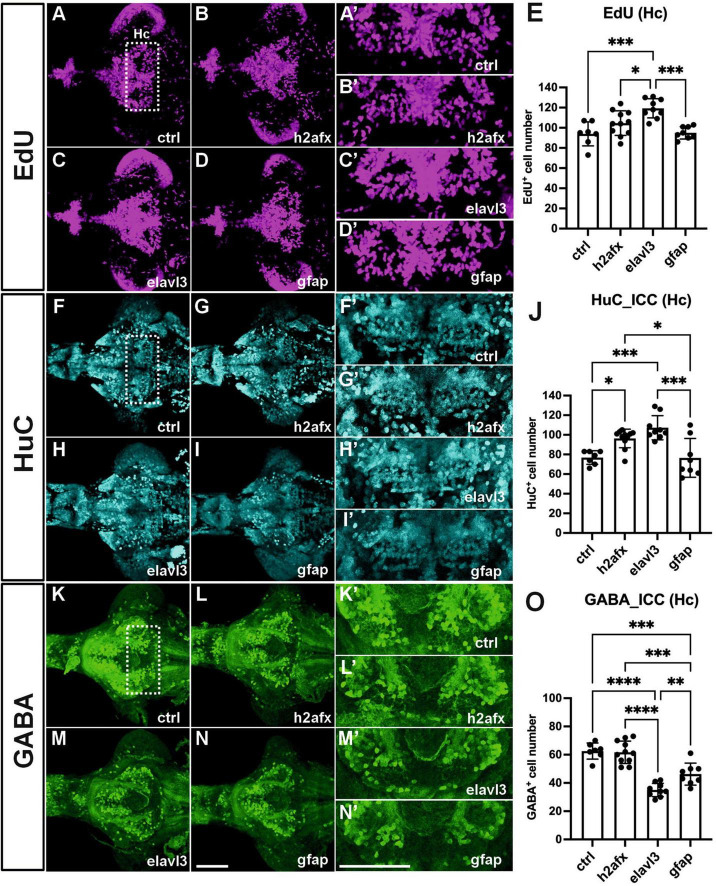
Transgenic expression of zebrafish *angpt1* increases proliferation and HuC-positive cells but reduces GABAergic neurons. 5-dpf dissected brains were collected from Turku wild-type embryos injected with Tol2 constructs, including control or *angpt1* transgene driven by *h2afx*, *elavl3*, and *gfap* promoter at the one-cell stage. Brain samples were stained with anti-HuC and anti-GABA antibodies following the EdU assay. EdU-proliferation images shown in **(A)** control **(B)** *h2afx*: *angpt1* **(C)** *elavl3*: *angpt1* **(D)** *gfap*: *angpt1* **(E)** Quantification of EdU-positive cell numbers in the Hc region [*N* = 7–11 per injected group, *F*(3, 31) = 10.75 *P* < 0.0001]. HuC-immunostaining images shown in **(F)** control **(G)** *h2afx*: *angpt1* **(H)** *elavl3*: *angpt1* **(I)** *gfap*: *angpt1* **(J)** Quantification of HuC-positive cell numbers in the Hc region [*N* = 7–11 per injected group, *F*(3, 31) = 11.58, *P* < 0.0001]. GABA-immunostaining images show **(K)** control **(L)** *h2afx*: *angpt1* **(M)** *elavl3*: *angpt1* **(N)** *gfap*: *angpt1* **(O)** Quantification of GABA-positive cell numbers in the Hc region [*N* = 7–11 per injected group, *F*(3, 31) = 33.92, *P* < 0.0001]. **(A’–N’)** Show the high magnification images corresponding to the white rectangular area in the Hc region shown in **(A)** and equivalent in **(B–N)**. EdU-positive cells are displayed in magenta, HuC-positive cells in cyan, and GABA-positive cells in green. Hc, caudal hypothalamus. Statistical analysis is shown in mean ± SD by an ordinary one-way ANOVA with the Tukey multiple comparisons test. *p < 0.05, **p < 0.01, ***p < 0.001 and ****p < 0.0001. Scale bar is 200 μm.

To identify critical cell types of overexpressing *angpt1* that would produce potent neurogenic effects on brain development, three transgenic constructs carrying zebrafish *angpt1* driven by the *h2afx* promoter (quasi-ubiquitous expression) (Kwan et al., 2007), the *elavl3* promoter (pan-neuronal expression) (Don et al., 2017), and the *gfap* promoter (radial glial and astrocyte expression) (Don et al., 2017), were injected in Turku WT embryos at the one-cell stage. 6-dpf dissected brains with conditionally overexpressed *angpt1* were co-stained with antibodies recognizing GABA (a GABAergic marker), and HuC/D (a pan-neuronal marker) following the EdU staining (a proliferation marker). The positively labeled cells stained with these markers in the caudal hypothalamus (Hc) were counted. The significant statistics is stated in the figure legend. We found a significant increase in proliferating cell numbers in the *elavl3*-driven *angpt1* group (Figures 7C, C’, E) compared with those of the control-injected (Figures 7A, A’), the *h2afx*-driven (Figures 7B, B’), and the *gfap*-driven *angpt1* groups (Figures 7D, D’). A significant increase in HuC-positive cell numbers was found in the *h2afx* -driven (Figures 7G, G’, J) and the *elavl3*-driven *angpt1* groups (Figures 7H, H’) compared with the control- injected (Figures 7F, F’) and the *gfap*-driven *angpt1* groups (Figures 7I, I’). In contrast to a considerable increase in proliferating cells and neuronal progenitors, a substantial decrease in GABA-positive cell numbers was found in the *elavl3*-driven *angpt1* (Figures 7M, M’, O) and in the *gfap*-driven *angpt1* groups (Figures 7N, N’, O) compared with the control-injected (Figures 7K, K’) and the *h2afx*-driven *angpt1* groups (Figures 7L, L’, O). These data indicate that overexpression of *angpt1* in neuronal precursors stimulates proliferation but has a downregulating effect on GABAergic neurons in the caudal hypothalamus area.

### 3.8 Neuronal overexpression of zebrafish *angpt1* in *tek*^–/–^ larval brains promotes proliferation and neuronal precursors in a *tek*-independent fashion

**FIGURE 8:**
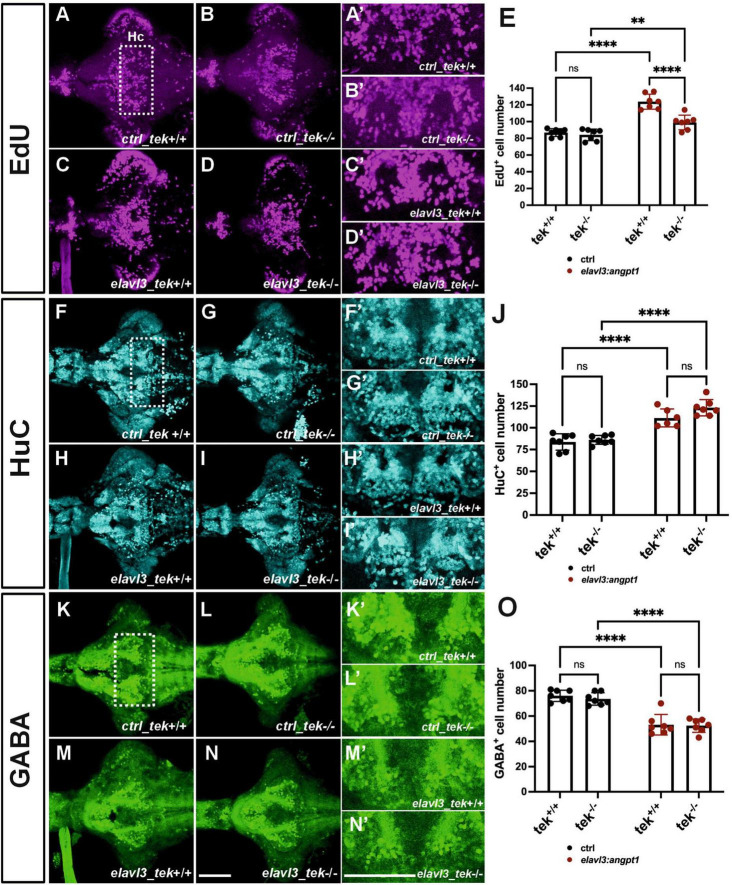
Zebrafish angpt1 regulates neurogenesis in a tek-independent manner. 5-dpf dissected brains were collected from *tek*^+/+^ and *tek*^–/–^ embryos injected with Tol2 constructs including control, *angpt1* transgene driven by *h2afx*, *elavl3*, or *gfap* promoter at one-cell stage. The brain samples were stained with anti-HuC and anti-GABA antibodies following the EdU assay. EdU-proliferation images shown in **(A)** control of *tek*^+/+^ **(B)** control of *tek*^–/–^ **(C)** *tg*(*elavl3*: *angpt1*) in *tek*^+/+^ **(D)** *tg*(*elavl3*: *angpt1*) in *tek*^–/–^ **(E)** Quantification of EdU-positive cell numbers in the Hc region (*N* = 7 per transgene-genotype group, genotype factor *F*(1, 24) = 23.43, *P* < 0.0001; transgene factor *F*(1, 24) = 82.89, *P* < 0.0001). HuC-immunostaining images show **(F)** control of *tek*^+/+^ **(G)** control of *tek*^–/–^ **(H)** *tg*(*elavl3*: *angpt1*) in *tek*^+/+^ **(I)** *tg*(*elavl3*: *angpt1*) in *tek*^–/–^ **(J)** Quantification of HuC-positive cell numbers in the Hc region [*N* = 7 per transgene-genotype group, genotype factor *F*(1, 23) = 4.501, *P* = 0.0449; transgene factor *F*(1, 23) = 92.70, *P* < 0.0001]. GABA-immunostaining images show **(K)** control of *tek*^+/+^ **(L)** control of *tek*^–/–^ **(M)** *tg*(*elavl3*: *angpt1*) in *tek*^+/+^ **(N)** *tg*(*elavl3*: *angpt1*) in *tek*^–/–^ **(O)** Quantification of GABA-positive cell numbers in the Hc region [*N* = 7–11 per transgene-genotype group, genotype factor *F*(1, 24) = 0.5009, *P* = 0.4859; transgene factor *F*(1, 24) = 98.17, *P* < 0.0001]. **(A’–N’)** Show the high magnification images corresponding to the white rectangular area in the Hc region shown in **(A)** and equivalent in **(B–N)**. EdU-positive cells in magenta, HuC-positive cells in cyan, and GABA-positive cells in green. Hc, caudal hypothalamus. Statistical analysis is shown in mean ± SD by an ordinary two-way ANOVA with Sidak’s multiple comparisons test, with a single pooled variance. **p < 0.01 and ****p < 0.0001. Scale bar is 200 μm.

Neuronal overexpression of *angpt1* substantially increased proliferation and progenitor cells in the Turku WT larval brains (Figure 7). To investigate whether *angpt1* and its primarily binding receptor *tek* equivalently contribute to the regulation of the embryogenic neurogenesis, the *tek*^+/+^ and *tek*^–/–^ larval fish is subjected to overexpression of *angpt1* driven by the *elavl3* promoter, and the proliferating and neuronal cell numbers are counted after immunostaining. A significant increase in the proliferating cells was found in the *elavl3*-driven *angpt1* injected *tek^+/+^* (Figures 8C, C’, E) and *tek*^–/–^ groups (Figures 8D, D’, E) compared with the control-injected *tek*^+/+^ (Figures 8A, A’) and *tek*^–/–^ (Figures 8B, B’) groups, respectively. Notably, the *elavl3*-driven *angpt1* injected *tek*^+/+^ group produced a more substantial effect on proliferation than the *elavl3*-driven *angpt1 tek*^–/–^ group (Figure 8E). Moreover, in comparison with their control-injected groups (Figures 8F, F’, G, G’, J), the number of HuC-positive cells was significantly increased in the *elavl3*-driven *angpt1* injected *tek*^+/+^ (Figures 8H, H’) and *tek*^–/–^ (Figures 8I, I’, J) groups. In contrast, a significant reduction in GABAergic neurons was found in the *elavl3*-driven *angpt1 tek*^+/+^ (Figures 8M, M’) and *tek*^–/–^ groups (Figures 8N, N’, O) compared with their control-injected groups (Figures 8K, K’, L, L’, O). Overexpression of angpt1 promoting the proliferation of neural progenitors in the *tek*^–/–^ brain provides direct evidence that angpt1 without the functional *tek* receptor has a neurogenic impact on modulating neurogenesis in the zebrafish brain.

## 4 Discussion

Our present data first show that *angpt1* expression is found in the *notch1a*-, *dla*- and *nestin*-expressing domains in the ventricular proliferative zones (Mueller and Wullimann, 2003). We further show that *angpt1* and *itgb1b* play essential roles in developing cerebrovascular formation and exert their neurogenic effects through notch and wnt signaling pathways on the patterning of hindbrain reticulospinal neurons and regulation of hypothalamic neurotransmitter populations, including dopaminergic, histaminergic, and GABAergic systems in brain development. Our findings also provide evidence that the role of tek is dispensable during embryonic neurogenesis in zebrafish, in agreement with the published results of the angiogenesis aspect (Gjini et al., 2011; Jiang et al., 2020).

Increasing evidence shows that after brain trauma, Angpt1 functions dually in promoting angiogenesis and neurogenesis to enhance BBB integrity and neurological regeneration (Meng et al., 2014; Michinaga and Koyama, 2019; Wang et al., 2019) in addition to its indispensable roles in the developmental cardiovascular formation and the maintenance of adult vascular stability. Moreover, in neural cell cultures, Angpt1 acts directly on neurite outgrowth via a beta1-integrin-dependent manner instead of binding to its typical receptor, Tek (Carlson et al., 2001; Chen X. et al., 2009). However, the molecular mechanisms of neurogenic effects of *angpt1* and *itgb1b* in normal neurodevelopment remain primarily unrevealed. The Angpt1 and Tek knockout mice die between E9.5 and E12.5 because of cardiovascular failure (Sato et al., 1995; Suri et al., 1996). Consequently, embryonic lethality makes it challenging to observe the role of Angpt1 and Tek in embryonic neurogenesis in mammals.

In this study, we take advantage of zebrafish fast *ex utero* development and individual neurotransmitter systems in the CNS already developed within 7dpf (Panula et al., 2010), which allows us to characterize the overt vascular formation and the evident neural phenotypes in *angpt1*^–/–^, *itgb1b*^–/–^, and *tek*^–/–^ brains during embryonic development. The *angpt1*^–/–^ and *itgb1b*^–/–^ fish showing severe neurodevelopmental and cardiovascular abnormalities exhibited lethality between 5 and 7 dpf. However, the *tek^–/–^* fish normally developed dissimilar to the lethal phenotype of the Tek knockout mice. Our neural *angpt1*-overexpressing approach also demonstrated the neurogenic effect of zebrafish *angpt1* on promoting the proliferation of neuronal progenitors in the developing brain, indicating the importance of the precise spatiotemporal distribution of angpt1 in controlling neurogenesis along with angiogenesis.

In zebrafish, embryonic neurogenesis starts at 10 hpf (early somitogenesis) (Schmidt et al., 2013), earlier than cerebral vessel sprouting at around 24 hpf (late somitogenesis) (Ulrich et al., 2011). We identified that zebrafish *angpt1* was detectable from the maternal stage, and the expression level was high compared with its receptors. The expression pattern was ubiquitously distributed in the ectoderm and mesoderm until the gastrula period and later restricted in the brain and vasculature. The *angpt1* mRNA was also found in the ventricular proliferative zones where some of the *angpt1*-positive signals were discovered in the *notch1a*-, *dla*- and *nestin*-expressing regions, indicating that angpt1 may act as a potent pro-neurogenic factor in regulating embryonic neurogenesis via the notch1 signaling pathway before its role in angiogenesis.

During development, the cerebrovasculature was severely impaired in the *angpt1*^–/–^ (this study) and *itgb1b*^–/–^ zebrafish (Iida et al., 2018), and the deficiency of proliferation but a robust increase of the gfap-positive cells was found in these two mutant brains. The gross phenotype was accompanied by a significant down-regulation of neurogenic genes, including *pcna*, *notch1a*, *nestin*, *wnt1*, *wnt10b*, *pax5*, and *sox2*. The notch1 signaling pathway is crucial in maintaining NSC proliferation and promoting radial glia precursors fates (Koch et al., 2013). It also significantly regulates cardiomyocyte proliferation and arterial-venous determination (Niessen and Karsan, 2008). Wnt pathways activate quiescent radical glial cells to committed neuronal progenitors, and its activity also involves regulating vascular development in the developing brain (Menet et al., 2020). When Notch and Wnt signaling perturbation occurs, radial glial cells pause at the symmetrical self-renewal state and fail to commit the subsequent steps (Schmidt et al., 2013). This will destroy neuronal progenitor differentiation into mature neurons, resulting in neurodevelopmental disorders. Therefore, the abnormality of notch1 and wnt expression produces a disruption from the proliferation to neuronal differentiation that may explain the cause of severe brain defects in these two mutants. Consistent with this current finding, the larger brain population in the guppy has a higher expression level of *angpt1* than the smaller brain population in a notch1-dependent manner (Chen et al., 2015).

Notch and wnt pathways are essential for establishing neurotransmitter circuits (Castelo-Branco et al., 2003; Mahler et al., 2010; Sundvik et al., 2013). The impaired neurogenesis resulted in a considerable decrease of dopaminergic and histaminergic neurons in the *angpt1* and *itgb1b* mutant brains. Notably, transgenic overexpression of zebrafish *angpt1* in pan-neuronal cells promoted a dramatic increase of proliferation and neuronal progenitors; in contrast, a decreased number of GABAergic neurons was found in these transgenic larval brains. A recent finding reports that *Angpt1* shows a most robust expression in cerebellar GABA interneuron progenitors in the developing cerebellum, indicating its role in regulating the proliferation and migration of interneuron progenitors (Wei et al., 2021). A decrease of GABAergic neurons found in the *angpt1*-deficient and *angpt1*-overpressing brain may be due to two aspects: the alternations of angpt1 expression cause disorganized angiogenesis that misleads GABAergic progenitors to the wrong direction and trigger neural apoptosis resulting in a reduction of GABAergic neurons (Licht and Keshet, 2015). During neurogenesis, the nascent vasculature offers not only trophic factors and certain signaling factors to drive neural proliferation and differentiation (Tan et al., 2016; Li et al., 2018), but it also serves as a guiding framework for newly born neurons migrating to the suitable locations (Won et al., 2013). On the other hand, ectopic expression of angpt1 associated with extensive proliferation may negatively decline the maturation of GABAergic progenitors, causing a decrease in the GABAergic neurons in the hypothalamus. This reveals the curial role of *angpt1*, *which* contributes to GABAergic neurons, although, in this study, we have no evidence to prove that *angpt1* is directly involved in regulating GABAergic development.

The hindbrain organization requires interdependence between genetic, morphological, and neuroanatomical segmentation (Moens and Prince, 2002). In zebrafish brain vascularization, the primordial hindbrain channels (PHBCs) arise at around 20 hpf, extending laterally along the anterior-posterior axis and contributing to hindbrain vascularization. The first cerebral angiogenic sprouts developing from the inner lateral side of both PHBCs integrate correspondingly into neuroepithelium alongside the rhombomere boundaries after 28 hpf (Fujita et al., 2011; Ulrich et al., 2011). In zebrafish hindbrain organization, Mauthner cells are the giant reticulospinal neurons, reside bilaterally in rhombomere 4 (r4), and are the first identified hindbrain neurons beginning at 8–10 hpf (during gastrulation). No later than 18 hpf, axonal projections penetrate the segments in a stereotyped manner before the neuromeres are formed at 18–20 hpf (Moens and Prince, 2002). Accordingly, the branchiomotor (BM) neurons of the cranial nerves and the reticulospinal (RS) interneurons develop in a rhombomere-specific disposition and provide markers of segmental identity (Moens and Prince, 2002). The specificity of each segment and the identity of rhombomere boundaries require transcription factors, angioneurins, and Wnt-delta-Notch signaling pathways to maintain rhombomeres’ specification (Riley et al., 2004). For instance, Krox-20, a zinc finger transcription factor, is required to develop rhombomeres 3 and 5 and maintain regional specifications. Loss of functional Krox-20 causes disorganized odd- and even-numbered territories alongside the hindbrain and neuronal organization corresponding to the rhombomeres (Schneider-Maunoury et al., 1993; Voiculescu et al., 2001). Angioneurins, secreted and transmembrane proteins such as VEGF and Eph-ephrin, empower vasculotrophic and neurotrophic activities (Xu et al., 2000; Schwarz et al., 2004). Impairment of these factors disrupts the regular segment-restricted expression of krox-20 in the zebrafish hindbrain (Giudicelli et al., 2001; Laussu et al., 2017). Wnt1 regulates neurogenesis and mediates lateral inhibition of boundary cell specification in the zebrafish hindbrain via Notch signaling pathways (Xu et al., 1995; Cooke et al., 2001; Amoyel et al., 2005). Since the neural organization occurs earlier than the vessel invasions in the hindbrain formation, in the *angpt1* and *itgb1b* mutant hindbrain, the deficiency of reticulospinal neurons accompanied by downregulation of *notch1a*, *wnt1*, and *krox20* renders it possible that angpt1 and itgb1b may function in angioneurin-like manners having neurotrophic effects regulating segment-specific fates and specifying rhombomeric territories even before angiogenesis proceeds.

## 5 Conclusion

In this study, we used both loss-of-function and gain-of-function approaches to provide novel evidence that angpt1 and itgb1b perform dual functions in zebrafish angiogenesis and embryonic neurogenesis for developing normal hindbrain morphogenesis and mainly promote neural proliferation in a *tek*-autonomous manner. Although this study did not demonstrate the direct interaction between angpt1 and itgb1b, the previous research showed overexpression of α5β1 integrins and angpt1 stimulating angiogenesis following ischemic stroke (Wang et al., 2019). In the upcoming studies, it would be beneficial for the preclinical study to generate cell-type/tissue-specific or conditional *angpt1* and *igtb1b* mutants to investigate the neurogenic effects of angpt1 and itgb1b in the later stages. Our findings highlight the essential neurogenic effects of angpt1 by mean of notch1 and wnt pathways and its putative receptor itgb1b that may support the concept of angiopoietin-based treatments for clinical therapeutics in neurological disorders such as Alzheimer’s disease, stroke, and traumatic brain injuries (Zlokovic, 2011; Venkat et al., 2021).

## Data availability statement

The datasets presented in this study can be found in online repositories. The names of the repository/repositories and accession number(s) can be found in this article/Supplementary material.

## Ethics statement

The animal studies were approved by the Regional Government of Southern Finland’s permits, in agreement with the European Convention’s ethical guidelines. The studies were conducted in accordance with the local legislation and institutional requirements. Written informed consent was obtained from the owners for the participation of their animals in this study.

## Author contributions

Y-CC: Conceptualization, Data curation, Writing–original draft, Writing–review and editing. TM: Data curation, Writing–review and editing. VM: Data curation, Writing–review and editing. PP: Conceptualization, Funding acquisition, Investigation, Writing–review and editing.

## Footnotes

1
https://shigen.nig.ac.jp/zebra/index_en.html

2
https://www.ncbi.nlm.nih.gov/tools/primer-blast

## References

[B1] Adams R. H. Eichmann A. (2010). Axon guidance molecules in vascular patterning. *Cold Spring Harb. Perspect. Biol.* 2:a001875. 10.1101/cshperspect.a001875 PMC2857165 20452960

[B2] Alexandre D. Clarke J. D. Oxtoby E. Yan Y. L. Jowett T. Holder N. (1996). Ectopic expression of Hoxa-1 in the zebrafish alters the fate of the mandibular arch neural crest and phenocopies a retinoic acid-induced phenotype. *Development* 122 735–746. 10.1242/dev.122.3.735 8631251

[B3] Amoyel M. Cheng Y. C. Jiang Y. J. Wilkinson D. G. (2005). Wnt1 regulates neurogenesis and mediates lateral inhibition of boundary cell specification in the zebrafish hindbrain. *Development* 132 775–785. 10.1242/dev.01616 15659486

[B4] Artavanis-Tsakonas S. Rand M. D. Lake R. J. (1999). Notch signaling: Cell fate control and signal integration in development. *Science* 284 770–776. 10221902 10.1126/science.284.5415.770

[B5] Aujla P. K. Bogdanovic V. Naratadam G. T. Raetzman L. T. (2015). Persistent expression of activated notch in the developing hypothalamus affects survival of pituitary progenitors and alters pituitary structure. *Dev. Dyn.* 244 921–934. 10.1002/dvdy.24283 25907274 PMC4520742

[B6] Bai Y. Cui M. Meng Z. Shen L. He Q. Zhang X. (2009). Ectopic expression of angiopoietin-1 promotes neuronal differentiation in neural progenitor cells through the Akt pathway. *Biochem. Biophys. Res. Commun.* 378 296–301. 10.1016/j.bbrc.2008.11.052 19028450

[B7] Carlson T. R. Feng Y. Maisonpierre P. C. Mrksich M. Morla A. O. (2001). Direct cell adhesion to the angiopoietins mediated by integrins. *J. Biol. Chem.* 276 26516–26525. 10.1074/jbc.M100282200 11346644

[B8] Carmeliet P. Tessier-Lavigne M. (2005). Common mechanisms of nerve and blood vessel wiring. *Nature* 436 193–200. 16015319 10.1038/nature03875

[B9] Castelo-Branco G. Wagner J. Rodriguez F. J. Kele J. Sousa K. Rawal N. (2003). Differential regulation of midbrain dopaminergic neuron development by Wnt-1, Wnt-3a, and Wnt-5a. *Proc. Natl. Acad. Sci. U.S.A.* 100 12747–12752. 10.1073/pnas.1534900100 14557550 PMC240689

[B10] Castets M. Mehlen P. (2010). Netrin-1 role in angiogenesis: To be or not to be a pro-angiogenic factor? *Cell Cycle* 9 1466–1471. 20372055 10.4161/cc.9.8.11197

[B11] Chen X. Fu W. Tung C. E. Ward N. L. (2009). Angiopoietin-1 induces neurite outgrowth of PC12 cells in a Tie2-independent, beta1-integrin-dependent manner. *Neurosci. Res.* 64 348–354. 10.1016/j.neures.2009.04.007 19379779 PMC2730485

[B12] Chen Y. C. Baronio D. Semenova S. Abdurakhmanova S. Panula P. (2020). Cerebral dopamine neurotrophic factor regulates multiple neuronal subtypes and behavior. *J. Neurosci.* 40 6146–6164. 32631936 10.1523/JNEUROSCI.2636-19.2020 PMC7406287

[B13] Chen Y. C. Harrison P. W. Kotrschal A. Kolm N. Mank J. E. Panula P. (2015). Expression change in Angiopoietin-1 underlies change in relative brain size in fish. *Proc. Biol. Sci.* 282:20150872. 10.1098/rspb.2015.0872 26108626 PMC4590489

[B14] Chen Y. C. Priyadarshini M. Panula P. (2009). Complementary developmental expression of the two tyrosine hydroxylase transcripts in zebrafish. *Histochem. Cell Biol.* 132 375–381. 19603179 10.1007/s00418-009-0619-8

[B15] Chen Y. C. Semenova S. Rozov S. Sundvik M. Bonkowsky J. L. Panula P. (2016). A novel developmental role for dopaminergic signaling to specify hypothalamic neurotransmitter identity. *J. Biol. Chem.* 291 21880–21892. 10.1074/jbc.M115.697466 27539857 PMC5063973

[B16] Cooke J. Moens C. Roth L. Durbin L. Shiomi K. Brennan C. (2001). Eph signalling functions downstream of Val to regulate cell sorting and boundary formation in the caudal hindbrain. *Development* 128 571–580. 10.1242/dev.128.4.571 11171340

[B17] Don E. K. Formella I. Badrock A. P. Hall T. E. Morsch M. Hortle E. (2017). A Tol2 gateway-compatible toolbox for the study of the nervous system and neurodegenerative disease. *Zebrafish* 14 69–72. 10.1089/zeb.2016.1321 27631880

[B18] Engler A. Zhang R. Taylor V. (2018). Notch and neurogenesis. *Adv. Exp. Med. Biol.* 1066 223–234. 30030829 10.1007/978-3-319-89512-3_11

[B19] Fujita M. Cha Y. R. Pham V. N. Sakurai A. Roman B. L. Gutkind J. S. (2011). Assembly and patterning of the vascular network of the vertebrate hindbrain. *Development* 138 1705–1715. 10.1242/dev.058776 21429985 PMC3074447

[B20] Gale N. W. Yancopoulos G. D. (1999). Growth factors acting via endothelial cell-specific receptor tyrosine kinases: VEGFs, angiopoietins, and ephrins in vascular development. *Genes Dev.* 13 1055–1066. 10323857 10.1101/gad.13.9.1055

[B21] Giudicelli F. Taillebourg E. Charnay P. Gilardi-Hebenstreit P. (2001). Krox-20 patterns the hindbrain through both cell-autonomous and non cell-autonomous mechanisms. *Genes Dev.* 15 567–580. 10.1101/gad.189801 11238377 PMC312642

[B22] Gjini E. Hekking L. H. Kuchler A. Saharinen P. Wienholds E. Post J. A. (2011). Zebrafish Tie-2 shares a redundant role with Tie-1 in heart development and regulates vessel integrity. *Dis. Model Mech.* 4 57–66. 10.1242/dmm.005033 21045210 PMC3014345

[B23] Guijarro-Munoz I. Cuesta A. M. Alvarez-Cienfuegos A. Geng J. G. Alvarez-Vallina L. Sanz L. (2012). The axonal repellent Slit2 inhibits pericyte migration: Potential implications in angiogenesis. *Exp. Cell Res.* 318 371–378. 10.1016/j.yexcr.2011.12.005 22198087

[B24] Hatakeyama M. Ninomiya I. Kanazawa M. (2020). Angiogenesis and neuronal remodeling after ischemic stroke. *Neural Regen. Res.* 15 16–19. 31535636 10.4103/1673-5374.264442 PMC6862417

[B25] Hauptmann G. Lauter G. Soll I. (2016). Detection and signal amplification in zebrafish RNA FISH. *Methods* 98 50–59. 26821229 10.1016/j.ymeth.2016.01.012

[B26] Hu Y. Xie S. Yao J. (2016). Identification of novel reference genes suitable for qRT-PCR normalization with respect to the zebrafish developmental stage. *PLoS One* 11:e0149277. 10.1371/journal.pone.0149277 26891128 PMC4758726

[B27] Huang S. Y. Feng C. W. Hung H. C. Chakraborty C. Chen C. H. Chen W. F. (2014). A novel zebrafish model to provide mechanistic insights into the inflammatory events in carrageenan-induced abdominal edema. *PLoS One* 9:e104414. 10.1371/journal.pone.0104414 25141004 PMC4139260

[B28] Iida A. Wang Z. Hirata H. Sehara-Fujisawa A. (2018). Integrin beta1 activity is required for cardiovascular formation in zebrafish. *Genes Cells* 23 938–951. 30151851 10.1111/gtc.12641

[B29] Jiang Z. Carlantoni C. Allanki S. Ebersberger I. Stainier D. Y. R. (2020). Tek (Tie2) is not required for cardiovascular development in zebrafish. *Development* 147:dev193029. 10.1242/dev.193029 32928907

[B30] Karhunen T. Airaksinen M. S. Tuomisto L. Panula P. (1993). Neurotransmitters in the nervous system of Macoma balthica (Bivalvia). *J. Comp. Neurol.* 334 477–488. 7690786 10.1002/cne.903340311

[B31] Kaslin J. Panula P. (2001). Comparative anatomy of the histaminergic and other aminergic systems in zebrafish (Danio rerio). *J. Comp. Neurol.* 440 342–377. 10.1002/cne.1390 11745628

[B32] Kawakami K. Takeda H. Kawakami N. Kobayashi M. Matsuda N. Mishina M. (2004). A transposon-mediated gene trap approach identifies developmentally regulated genes in zebrafish. *Dev. Cell* 7 133–144. 15239961 10.1016/j.devcel.2004.06.005

[B33] Kimmel C. B. Ballard W. W. Kimmel S. R. Ullmann B. Schilling T. F. (1995). Stages of embryonic development of the zebrafish. *Dev. Dyn*. 203, 253–310. 10.1002/aja.1002030302 8589427

[B34] Koch U. Lehal R. Radtke F. (2013). Stem cells living with a Notch. *Development* 140 689–704. 23362343 10.1242/dev.080614

[B35] Koh G. Y. (2013). Orchestral actions of angiopoietin-1 in vascular regeneration. *Trends Mol. Med.* 19 31–39. 10.1016/j.molmed.2012.10.010 23182855

[B36] Kukko-Lukjanov T. K. Panula P. (2003). Subcellular distribution of histamine, GABA and galanin in tuberomamillary neurons in vitro. *J. Chem. Neuroanat.* 25 279–292. 10.1016/s0891-0618(03)00043-7 12842273

[B37] Kwan K. M. Fujimoto E. Grabher C. Mangum B. D. Hardy M. E. Campbell D. S. (2007). The Tol2kit: A multisite gateway-based construction kit for Tol2 transposon transgenesis constructs. *Dev. Dyn.* 236 3088–3099. 10.1002/dvdy.21343 17937395

[B38] Laussu J. Audouard C. Kischel A. Assis-Nascimento P. Escalas N. Liebl D. J. (2017). Eph/Ephrin signaling controls progenitor identities in the ventral spinal cord. *Neural Dev.* 12:10. 10.1186/s13064-017-0087-0 28595615 PMC5463316

[B39] Li S. Kumar T. P. Joshee S. Kirschstein T. Subburaju S. Khalili J. S. (2018). Endothelial cell-derived GABA signaling modulates neuronal migration and postnatal behavior. *Cell Res.* 28 221–248. 10.1038/cr.2017.135 29086765 PMC5799810

[B40] Licht T. Keshet E. (2015). The vascular niche in adult neurogenesis. *Mech. Dev.* 138(Pt. 1), 56–62. 26103548 10.1016/j.mod.2015.06.001

[B41] Lin R. Cai J. Kenyon L. Iozzo R. Rosenwasser R. Iacovitti L. (2019). Systemic factors trigger vasculature cells to drive notch signaling and neurogenesis in neural stem cells in the adult brain. *Stem Cells* 37 395–406. 10.1002/stem.2947 30431198 PMC7028145

[B42] Lowery L. A. Sive H. (2004). Strategies of vertebrate neurulation and a re-evaluation of teleost neural tube formation. *Mech. Dev.* 121 1189–1197. 10.1016/j.mod.2004.04.022 15327780

[B43] Machluf Y. Gutnick A. Levkowitz G. (2011). Development of the zebrafish hypothalamus. *Ann. N. Y. Acad. Sci.* 1220 93–105. 21388407 10.1111/j.1749-6632.2010.05945.x

[B44] Mahler J. Driever W. (2007). Expression of the zebrafish intermediate neurofilament Nestin in the developing nervous system and in neural proliferation zones at postembryonic stages. *BMC Dev. Biol.* 7:89. 10.1186/1471-213X-7-89 17651502 PMC1950091

[B45] Mahler J. Filippi A. Driever W. (2010). DeltaA/DeltaD regulate multiple and temporally distinct phases of notch signaling during dopaminergic neurogenesis in zebrafish. *J. Neurosci.* 30 16621–16635. 10.1523/JNEUROSCI.4769-10.2010 21148001 PMC6634882

[B46] Melani M. Weinstein B. M. (2010). Common factors regulating patterning of the nervous and vascular systems. *Annu. Rev. Cell Dev. Biol.* 26 639–665. 19575651 10.1146/annurev.cellbio.093008.093324

[B47] Menet R. Lecordier S. Elali A. (2020). Wnt pathway: An emerging player in vascular and traumatic mediated brain injuries. *Front. Physiol.* 11:565667. 10.3389/fphys.2020.565667 33071819 PMC7530281

[B48] Meng Z. Li M. He Q. Jiang S. Zhang X. Xiao J. (2014). Ectopic expression of human angiopoietin-1 promotes functional recovery and neurogenesis after focal cerebral ischemia. *Neuroscience* 267 135–146. 10.1016/j.neuroscience.2014.02.036 24607344

[B49] Michinaga S. Koyama Y. (2019). Dual roles of astrocyte-derived factors in regulation of blood-brain barrier function after brain damage. *Int. J. Mol. Sci.* 20:571. 10.3390/ijms20030571 30699952 PMC6387062

[B50] Moens C. B. Prince V. E. (2002). Constructing the hindbrain: Insights from the zebrafish. *Dev. Dyn.* 224 1–17. 11984869 10.1002/dvdy.10086

[B51] Mosimann C. Kaufman C. K. Li P. Pugach E. K. Tamplin O. J. Zon L. I. (2011). Ubiquitous transgene expression and Cre-based recombination driven by the ubiquitin promoter in zebrafish. *Development* 138 169–177. 10.1242/dev.059345 21138979 PMC2998170

[B52] Mueller T. Wullimann M. F. (2003). Anatomy of neurogenesis in the early zebrafish brain. *Brain Res. Dev. Brain Res.* 140 137–155. 12524185 10.1016/s0165-3806(02)00583-7

[B53] Mulligan K. A. Cheyette B. N. (2012). Wnt signaling in vertebrate neural development and function. *J. Neuroimmune Pharmacol.* 7 774–787. 23015196 10.1007/s11481-012-9404-x PMC3518582

[B54] Niessen K. Karsan A. (2008). Notch signaling in cardiac development. *Circ. Res.* 102 1169–1181. 18497317 10.1161/CIRCRESAHA.108.174318

[B55] Paavola J. Schliffke S. Rossetti S. Kuo I. Y. Yuan S. Sun Z. (2013). Polycystin-2 mutations lead to impaired calcium cycling in the heart and predispose to dilated cardiomyopathy. *J. Mol. Cell Cardiol.* 58 199–208. 10.1016/j.yjmcc.2013.01.015 23376035 PMC3636149

[B56] Panula P. Chen Y. C. Priyadarshini M. Kudo H. Semenova S. Sundvik M. (2010). The comparative neuroanatomy and neurochemistry of zebrafish CNS systems of relevance to human neuropsychiatric diseases. *Neurobiol. Dis.* 40 46–57. 10.1016/j.nbd.2010.05.010 20472064

[B57] Riley B. B. Chiang M. Y. Storch E. M. Heck R. Buckles G. R. Lekven A. C. (2004). Rhombomere boundaries are Wnt signaling centers that regulate metameric patterning in the zebrafish hindbrain. *Dev. Dyn.* 231 278–291. 10.1002/dvdy.20133 15366005

[B58] Rosa A. I. Goncalves J. Cortes L. Bernardino L. Malva J. O. Agasse F. (2010). The angiogenic factor angiopoietin-1 is a proneurogenic peptide on subventricular zone stem/progenitor cells. *J. Neurosci.* 30 4573–4584. 10.1523/JNEUROSCI.5597-09.2010 20357108 PMC6632326

[B59] Sallinen V. Torkko V. Sundvik M. Reenila I. Khrustalyov D. Kaslin J. (2009). MPTP and MPP+ target specific aminergic cell populations in larval zebrafish. *J. Neurochem.* 108 719–731. 10.1111/j.1471-4159.2008.05793.x 19046410

[B60] Sato T. N. Tozawa Y. Deutsch U. Wolburg-Buchholz K. Fujiwara Y. Gendron-Maguire M. (1995). Distinct roles of the receptor tyrosine kinases Tie-1 and Tie-2 in blood vessel formation. *Nature* 376 70–74. 7596437 10.1038/376070a0

[B61] Schindelin J. Arganda-Carreras I. Frise E. Kaynig V. Longair M. Pietzsch T. (2012). Fiji: An open-source platform for biological-image analysis. *Nat. Methods* 9 676–682. 10.1038/nmeth.2019 22743772 PMC3855844

[B62] Schmidt R. Strahle U. Scholpp S. (2013). Neurogenesis in zebrafish - from embryo to adult. *Neural Dev.* 8:3. 10.1186/1749-8104-8-3 PMC3598338 23433260

[B63] Schmitt C. E. Holland M. B. Jin S. W. (2012). Visualizing vascular networks in zebrafish: An introduction to microangiography. *Methods Mol. Biol.* 843 59–67. 10.1007/978-1-61779-523-7_6 22222521

[B64] Schmittgen T. D. Livak K. J. (2008). Analyzing real-time PCR data by the comparative C(T) method. *Nat. Protoc.* 3 1101–1108. 10.1038/nprot.2008.73 18546601

[B65] Schneider-Maunoury S. Topilko P. Seitandou T. Levi G. Cohen-Tannoudji M. Pournin S. (1993). Disruption of Krox-20 results in alteration of rhombomeres 3 and 5 in the developing hindbrain. *Cell* 75 1199–1214. 10.1016/0092-8674(93)90329-o 7903221

[B66] Schwarz Q. Gu C. Fujisawa H. Sabelko K. Gertsenstein M. Nagy A. (2004). Vascular endothelial growth factor controls neuronal migration and cooperates with Sema3A to pattern distinct compartments of the facial nerve. *Genes Dev.* 18 2822–2834. 10.1101/gad.322904 15545635 PMC528901

[B67] Sundvik M. Chen Y. C. Panula P. (2013). Presenilin1 regulates histamine neuron development and behavior in zebrafish, danio rerio. *J. Neurosci.* 33 1589–1597. 10.1523/JNEUROSCI.1802-12.2013 23345232 PMC6618731

[B68] Suri C. Jones P. F. Patan S. Bartunkova S. Maisonpierre P. C. Davis S. (1996). Requisite role of angiopoietin-1, a ligand for the TIE2 receptor, during embryonic angiogenesis. *Cell* 87 1171–1180. 8980224 10.1016/s0092-8674(00)81813-9

[B69] Tan X. Liu W. A. Zhang X. J. Shi W. Ren S. Q. Li Z. (2016). Vascular influence on ventral telencephalic progenitors and neocortical interneuron production. *Dev. Cell* 36 624–638. 10.1016/j.devcel.2016.02.023 27003936 PMC4806403

[B70] Thisse C. Thisse B. (2008). High-resolution in situ hybridization to whole-mount zebrafish embryos. *Nat. Protoc.* 3 59–69. 18193022 10.1038/nprot.2007.514

[B71] Thomas J. L. Baker K. Han J. Calvo C. Nurmi H. Eichmann A. C. (2013). Interactions between VEGFR and Notch signaling pathways in endothelial and neural cells. *Cell Mol. Life Sci.* 70 1779–1792. 23479133 10.1007/s00018-013-1312-6 PMC3648205

[B72] Ulrich F. Ma L. H. Baker R. G. Torres-Vazquez J. (2011). Neurovascular development in the embryonic zebrafish hindbrain. *Dev. Biol.* 357 134–151. 21745463 10.1016/j.ydbio.2011.06.037

[B73] Venkat P. Ning R. Zacharek A. Culmone L. Liang L. Landschoot-Ward J. (2021). Treatment with an Angiopoietin-1 mimetic peptide promotes neurological recovery after stroke in diabetic rats. *CNS Neurosci. Ther.* 27 48–59. 10.1111/cns.13541 33346402 PMC7804913

[B74] Voiculescu O. Taillebourg E. Pujades C. Kress C. Buart S. Charnay P. (2001). Hindbrain patterning: Krox20 couples segmentation and specification of regional identity. *Development* 128 4967–4978. 11748134 10.1242/dev.128.24.4967

[B75] Walchli T. Wacker A. Frei K. Regli L. Schwab M. E. Hoerstrup S. P. (2015). Wiring the vascular network with neural cues: A CNS perspective. *Neuron* 87 271–296. 10.1016/j.neuron.2015.06.038 26182414

[B76] Wang L. Zhang X. Liu X. Feng G. Fu Y. Milner R. (2019). Overexpression of alpha5beta1 integrin and angiopoietin-1 co-operatively promote blood-brain barrier integrity and angiogenesis following ischemic stroke. *Exp. Neurol.* 321:113042. 10.1016/j.expneurol.2019.113042 31445044

[B77] Ward N. L. Lamanna J. C. (2004). The neurovascular unit and its growth factors: Coordinated response in the vascular and nervous systems. *Neurol. Res.* 26 870–883. 15727271 10.1179/016164104X3798

[B78] Wei X. Jessa S. Kleinman C. L. Phoenix T. N. (2021). Mapping angiopoietin1 expression in the developing and adult brain. *Dev. Neurosci.* 43 321–334. 10.1159/000518351 34348288

[B79] Won C. Lin Z. Kumar T. P. Li S. Ding L. Elkhal A. (2013). Autonomous vascular networks synchronize GABA neuron migration in the embryonic forebrain. *Nat. Commun.* 4:2149. 10.1038/ncomms3149 23857367 PMC3763945

[B80] Xie Y. Dorsky R. I. (2017). Development of the hypothalamus: Conservation, modification and innovation. *Development* 144 1588–1599. 10.1242/dev.139055 28465334 PMC5450842

[B81] Xu Q. Alldus G. Holder N. Wilkinson D. G. (1995). Expression of truncated Sek-1 receptor tyrosine kinase disrupts the segmental restriction of gene expression in the Xenopus and zebrafish hindbrain. *Development* 121 4005–4016. 10.1242/dev.121.12.4005 8575301

[B82] Xu Q. Mellitzer G. Wilkinson D. G. (2000). Roles of Eph receptors and ephrins in segmental patterning. *Philos. Trans. R. Soc. Lond. B Biol. Sci.* 355 993–1002. 11128993 10.1098/rstb.2000.0635 PMC1692797

[B83] Zlokovic B. V. (2011). Neurovascular pathways to neurodegeneration in Alzheimer’s disease and other disorders. *Nat. Rev. Neurosci.* 12 723–738. 22048062 10.1038/nrn3114 PMC4036520

