# Supplementary material for: Angiopoietin 1 and integrin beta 1b are vital for zebrafish brain development
Source: Front Cell Neurosci. 2024 Jan 3;17:1289794. doi: 10.3389/fncel.2023.1289794 (PMC10792015; doi:10.3389/fncel.2023.1289794)
Supplement: Supplementary file 1 [file Data_Sheet_1.pdf]

Supplementary data

Material and methods

Heart and blood circulation rate measurement

2-dpf *angpt1* KO and WT embryos were anesthetized with 0.0168% (w/v) tricaine (MS-222, Sigma) and mounted on 3% methylcellulose (Sigma, M0262) for imaging. Heart and blood circulation videos were recorded using an Olympus IX70 microscope (Olympus, Tokyo, Japan) and a Hamamatsu ORCA-Flash 4.0 CMOS camera with HCImage software (Hamamatsu Photonics, Shizuoka, Japan). The recording speed was 50 fps or 133 fps for heartbeats and blood circulation, respectively. Videos were analyzed frame by frame with ImageJ. The heart rate was quantified using manual counting by a blinded tester, and the blood circulation velocity was determined by tracking the flow of red blood cells based on Paavola et al. (Paavola et al., 2013).

Hemoglobin staining

Dechorionated embryos were incubated in the o-dianisidine solution (0.6mg/ml of o-dianisidine; D1943, Sigma; 10 mM sodium acetate pH4.5; 0.65% H_2O_2; 40% ethanol) in the dark for 30 min at RT. The stained embryos were washed with ddH_2O 3 times for 10 min and fixed in 4% PFA for two hours at RT. Pigments were removed by incubation in the bleaching solution containing 0.8% KOH, 0.9% H_2O_2, and 0.1% Tween-20. The stained samples were washed with 1xPBST 4 times for 15 min and embedded in 50% glycerol/ PBS for microscopy (Huang et al., 2014).

Microangiography

Microangiography was performed on 3-dpf *angpt1* and *itgb1b* mutant embryos. Anesthetized embryos were placed on a 1% agarose injection stage. Approximately 1.5 nl of the solution containing 2mg/ml of fluorescein isothiocyanate-dextran 2000kDa (FITC-Dextran 2000, Sigma 52471) was injected into the sinus venosa of anesthetized embryos (Schmitt et al., 2012). Injected embryos were embedded in 2% low-melting agarose and live imaged by confocal microscopy within 20 min after injections.

Supplementary Figure 1. Spatiotemporal expression of zebrafish angiogenic factors, *angpt1, angpt2a, angpt2b, tie1 and tek.* Expression patterns of angiogenic factors from 7 developing stages: (A) one-cell stage, (B) 50% epiboly, (C) bud, (D) head of prim-5, (E) trunk of prim-5, (F) long-pec and (G) protruding mouth (hatching period) by the whole-mount *in situ* hybridization.

Arrowheads indicate that angiogenic factors appear in blastoderm (BD), ectoderm/mesoderm (ED/MD), yolk, and yolk syncytial layer (YSL). From pharyngula to the hatching period, arrows display that signal appears in eyes (E), heart (H), liver (L), midbrain-hindbrain boundary (MHB), dorsal aorta (DA), branchial arch (AA), and posterior cardinal vein (PCV). Scale bar is 200 μm.

Supplementary Figure 2. Genotype and phenotype of angpt1 $^{-/-}$ larvae. Aberrant embryonic brain and cardiovascular development of *angpt1* KO embryos at 48 hpf. (A) A scheme of angpt1 protein domains containing a signal peptide, coiled-coil, and fibrinogen C-terminal domains. The wild-type sibling and *angpt1*^{sa14266} mutant sequence chromatograms show that the *angpt1*^{sa14264} KO allele carries a nonsense mutation resulting in Q261 to a stop codon (*) in the coiled-coil domain. The HRM genotype of 3-dpf tail clips of a*ngpt1*^{sa14264} embryos is produced by inbreeding heterozygous *angpt1*$^{+/sa14264}$ parents. The high-resolution melting curve analysis distinguishes wild-type *angpt1*$^{+/+}$ (WT), heterozygous *angpt1*$^{+/-}$ (HET) from homozygous a*ngpt1*$^{-/-}$ (KO) embryos. (B) Brightfield images show the a*ngpt1*$^{-/-}$ embryos display cardiac edema at 72 hpf. The 3-dpf a*ngpt1*$^{-/-}$ embryos show severe cardiac edema and smaller eye, head, and body length size compared with the wild-type-like siblings. (C) Quantification of heartbeats in min (hb) and (D) the average velocity of blood circulation (bc) at 48 hpf. (E) Velocity profiles of the blood cells circulating in the dorsal aorta of a*ngpt1*$^{+/+}$ and a*ngpt1*$^{-/-}$ embryos at 48hpf. (F) Quantification of body length, (G) brain size, and (H) eye size of the a*ngpt1*$^{+/+}$ siblings and a*ngpt1*$^{-/-}$ embryos at 3 dpf. The arrow indicates pericardial edema (pe). Sample numbers are shown in the graphs. Data are shown in mean ± SEM. ***$p<0.001$ by Student's t-test. Scale bar is 200 μm.

Supplementary Figure 3. Brain haemorrhage and impairments of cerebrovascular development in *angpt1* KO embryos. (A) The blood phenotype of 3-dpf a*ngpt1*$^{-/-}$ embryos was shown by whole mount *o*-dianisidine staining. Lateral views and ventral views show that a*ngpt1*$^{-/-}$ embryos lack hemoglobinized erythrocytes in the heart (arrow) and have impaired blood circulation with the accumulation of blood cells in the aorta and ventral tail (rectangle) and brain haemorrhage (dash line rectangle). (B) Microangiography of the vascular formation in *angpt1*$^{-/-}$ and its *angpt1*$^{+/+}$ siblings. Microangiography was done by injecting fluorescent dye directly into the sinus venosus in 3-dpf genotyped embryos. Lateral and dorsal views of maximal intensity projections of confocal z-stacks images indicate that loss of *angpt1* causes the malformation of the forebrain and hindbrain vascular patterning (white arrows indicate the vascular malformation in the angpt1-/-

compared with the angpt1+/+). (C) There are no apparent defects in the trunk vasculature of a*ngpt1*$^{-/-}$ embryos. CA, caudal artery; CtA, central artery; CV, caudal vein; DLAV, dorsal longitudinal anastomotic vessels; H, heart; MCeV, middle cerebral vein; PHBC, primordial hindbrain channel; Se, intersegmental vessel; SIV, subintestinal vein; VA/AA, ventral aorta/ branchial artery. CA, caudal artery; CCV, common cardinal vein; CV, caudal vein; DLAV, dorsal longitudinal anastomotic vessel; H, heart; PHS, primary head sinus. Scale bar is 200 μm.
