# Supplementary figures and images for: Angiopoietin 1 and integrin beta 1b are vital for zebrafish brain development
Source: Front Cell Neurosci. 2024 Jan 3;17:1289794. doi: 10.3389/fncel.2023.1289794 (PMC10792015; doi:10.3389/fncel.2023.1289794)

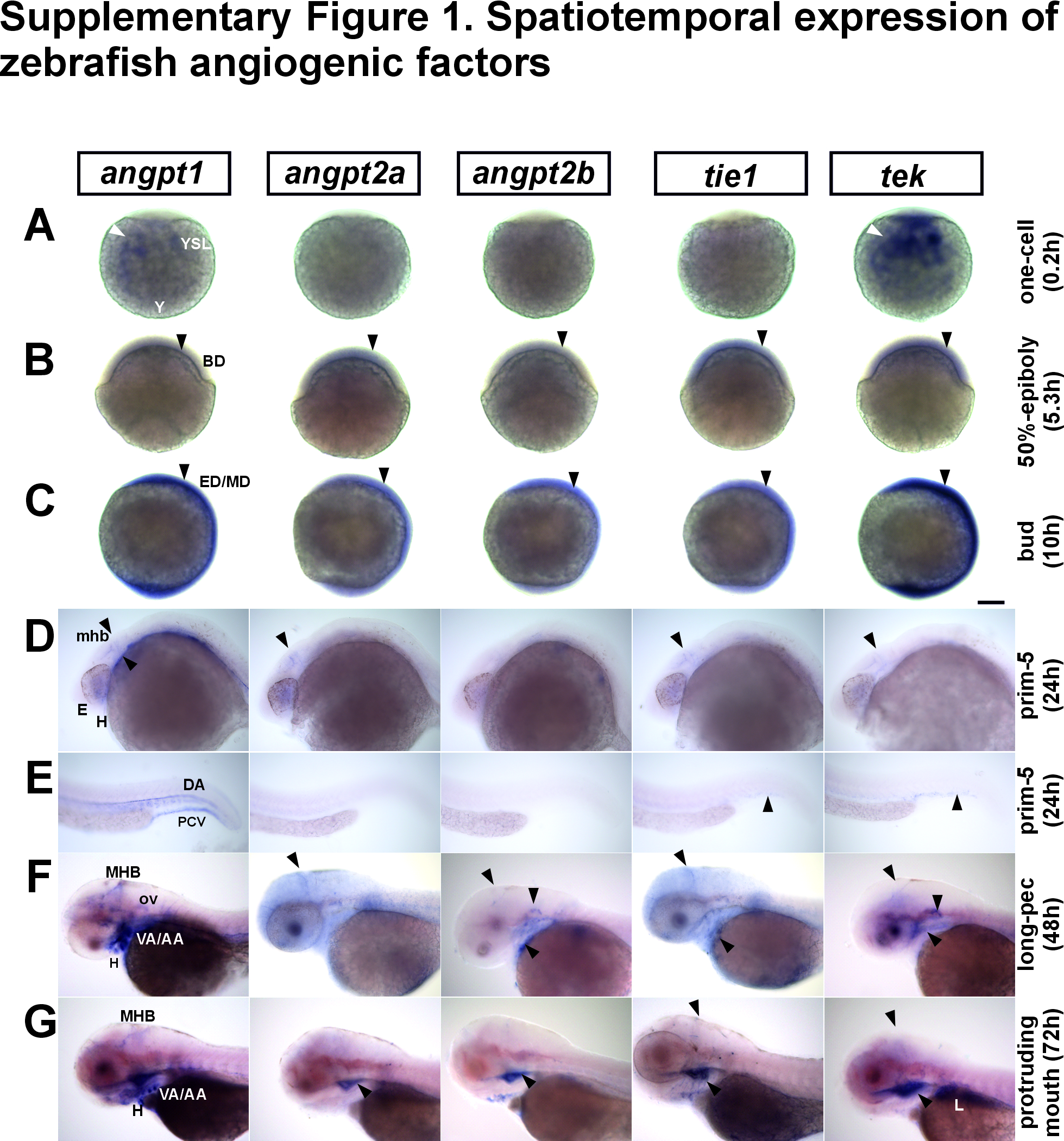

Supplement: Supplementary file 2 [file Image_1.jpeg]

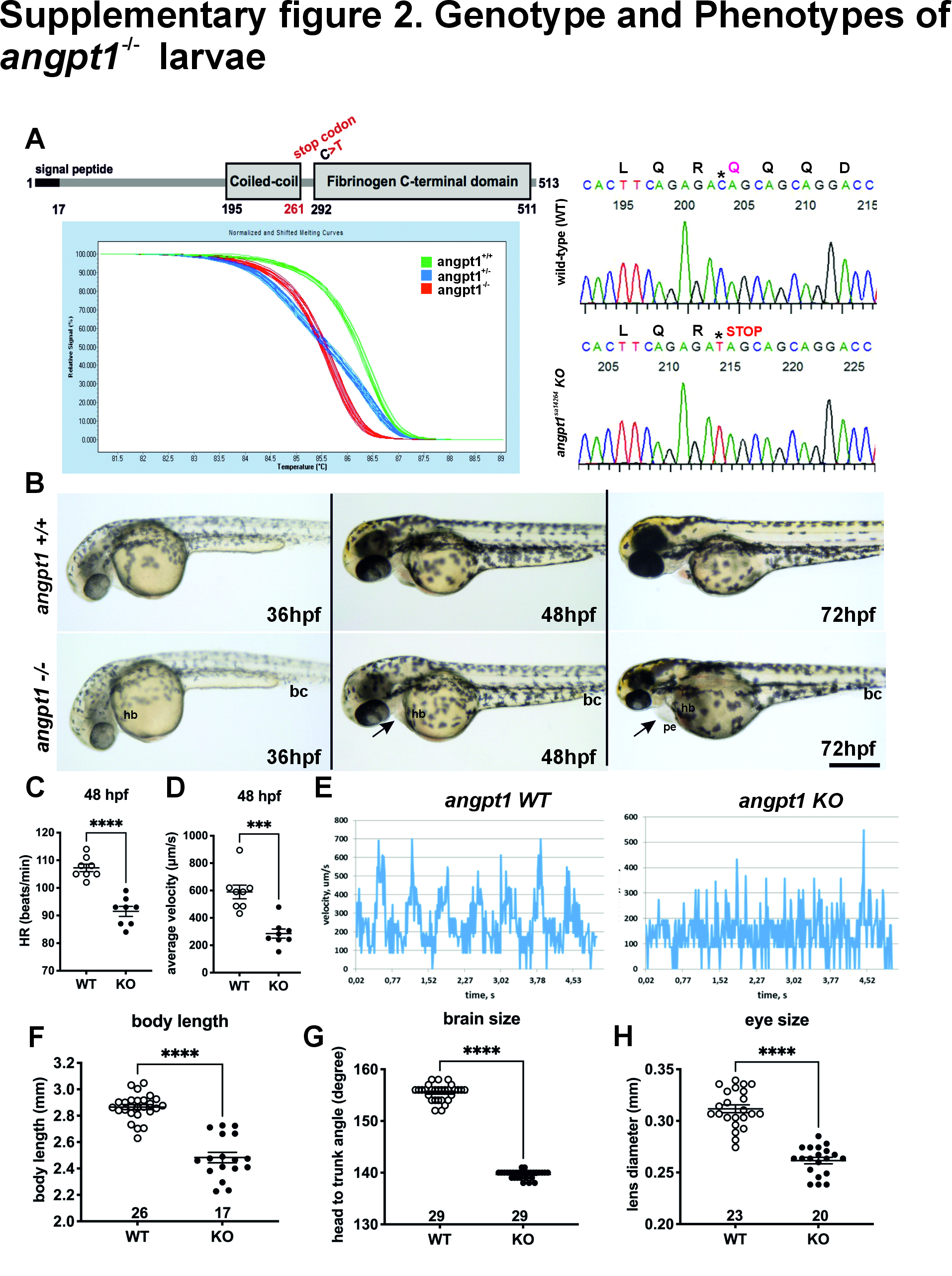

Supplement: Supplementary file 3 [file Image_2.jpeg]

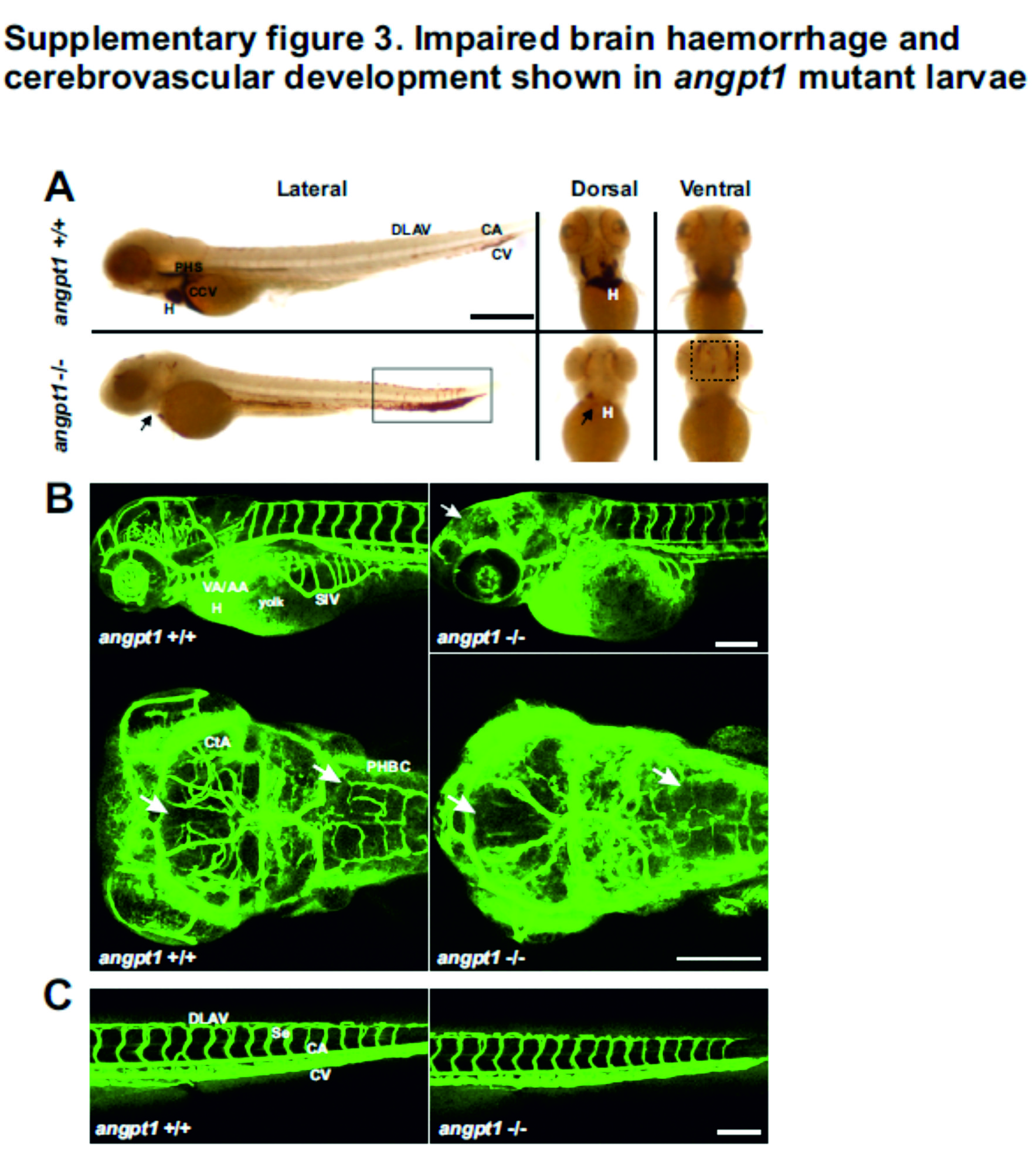

Supplement: Supplementary file 4 [file Image_3.jpeg]
